# Mechanosensor YAP mediates bone remodeling via NF-κB p65 induced osteoclastogenesis during orthodontic tooth movement

**DOI:** 10.1186/s40510-024-00548-w

**Published:** 2025-01-02

**Authors:** Jie Deng, Yu-Ning Zhang, Ru-Shui Bai, Ting-Ting Yu, Yi Zhao, Hao Liu, Yun-Fan Zhang, Tian-Min Xu, Bing Han

**Affiliations:** 1 Department of Orthodontics, Peking University School and Hospital of Stomatology & National Center of Stomatology & National Clinical Research Center for Oral Diseases & National Engineering Laboratory for Digital and Material Technology of Stomatology & Beijing Key Laboratory for Digital Stomatology & Research Center of Engineering and Technology for Computerized Dentistry Ministry of Health & NMPA Key Laboratory for Dental Materials, Beijing, China; 2 Department of Orthodontics, Nanjing Stomatological Hospital, Affiliated Hospital of Medical School, Institute of Stomatology, Nanjing University, Najing, China; 3 Cranial-Facial Growth and Development Center, Peking University School and Hospital of Stomatology, Beijing, China

**Keywords:** Yes-associated protein, Orthodontic tooth movement, Mechanotransduction, Bone remodeling, Osteoclast, NF-κB pathway

## Abstract

**Background:**

Yes-associated protein (YAP) is a crucial mechanosensor involved in mechanotransduction, but its role in regulating mechanical force-induced bone remodeling during orthodontic tooth movement (OTM) is unclear. This study aims to elucidate the relationship between mechanotransduction and mechanical force-induced alveolar bone remodeling during OTM.

**Results:**

Our study confirms an asynchronous (temporal and spatial sequence) remodeling pattern of the alveolar bone under mechanical force during OTM. Both compression and tension activate osteoclasts recruiting to the alveolar bone, whereas no significant presence of osteoblasts in the alveolar bone at the early stages of bone remodeling. Specifically, applying different force magnitudes (10, 25, 50, 100 g) to rats’ 1st molars affected OTM distance. Force-induced alveolar bone remodeling was characterized by osteoclastogenesis and YAP activation at compressive/tensile sites on day 1 of OTM. Notably, 25 g force triggered peak YAP expression and osteoclastic activity early on. Time-course analysis revealed two YAP activity peaks on day1 and 14, contrasting with one peak of type I collagen expression on day14. In addition, RNA-sequencing highlighted increased nuclear factor kappa B (NF-κB) signaling, mineral absorption, and osteoclast differentiation at day-1 and 3. Moreover, gene expression analysis showed similar trends for NF-κB p65, YAP1, and TEA domain 1 (TEAD1) during this time. Furthermore, experiments on osteoclast cultures indicated YAP activation via large tumor suppressor (LATS) and TEAD under mechanical stimuli (compression/tension), promoting osteoclastogenesis by regulating NF-κB p65 and receptor activator of NF-κB (RANK). Inhibiting YAP with verteporfin delayed OTM by impairing force-induced osteoclastic activities in vivo and ex-vivo.

**Conclusions:**

We propose that YAP mediates alveolar bone remodeling through NF-κB p65-induced osteoclastogenesis in an asynchronous remodeling pattern during OTM. Both compression and tension activate osteoclasts recruiting to the alveolar bone at early stages of bone remodeling, offering evidence for orthodontists as a reference.

**Supplementary Information:**

The online version contains supplementary material available at 10.1186/s40510-024-00548-w.

## Introduction

Orthodontic tooth movement (OTM) involves an orchestrated interplay between mechanotransduction and cellular response, with the alveolar bone remodeling process considered to work as the cornerstone of this dynamic process [1]. At its core lies the delicate balance between osteoclast-mediated bone resorption and osteoblast-driven bone formation [2–5]. Physiologically, bone remodeling, beginning with osteoclast-mediated bone resorption followed by osteoblast-driven bone formation, takes place asynchronously at anatomically distinct sites called basic multicellular units (BMUs), which are local teams of osteoclasts (OCs), osteoblasts (OBs), and reversal cells recently proven identical with osteoprogenitors [6]. Under mechanical stimulus, force exerts on the teeth during orthodontic treatment elicits cellular responses within the periodontal ligament (PDL) and adjacent alveolar bone (AB), initiating a cascade of molecular events. On the compression side, increased pressure stimulates OCs, leading to bone resorption and facilitating tooth movement. Conversely, on the tension side, OBs are activated with high expression levels of type I collagen (Col I) eventually, promoting bone deposition to maintain structural integrity [7]. OBs can mediate osteoclastogenesis via nuclear factor kappa B and its ligand (RANK/RANKL), while osteoprotegerin (OPG) is a soluble RANKL decoy receptor that is predominantly produced by OBs and which prevents OC formation and osteoclastic bone resorption by inhibiting the RANK/RANKL interaction [8]. However, it remains uncertain whether alveolar bone resorption and formation occur synchronously (or asynchronously) at the compressive and tensile sites during OTM under mechanical stimuli.

Previously, our research has demonstrated that both mechanical force-induced alveolar bone remodeling in rats and mechanical force-induced maxillofacial suture remodeling in rabbits exhibit early osteoclastogenesis [9–11]. In experimental OTM in rats, a substantial number of tartrate-resistant acid phosphatase (TRAP)-positive osteoclasts (TRAP^+^ OCs) and increased inflammation were observed at both compressive and tensile sites of roots, PDL, and adjacent alveolar bone within 1 to 3 days of force application [11]. Additionally, expressions of Cathepsin K (Ctsk) and RANKL were significantly upregulated in these areas shortly after mechanical loading [11]. These findings highlight the rapid onset of OC activity and inflammatory response following mechanical stimuli at both compressive and tensile sites, underscoring the early events in bone remodeling during OTM.

Mechanosensor yes-associated protein (YAP) is a transcriptional co-activator-associated protein of the hippo signaling pathway [12]. It binds to a transcriptional coactivator with a PDZ-binding motif (TAZ), as nuclear relays of mechanical signals, which is responsible for sensing mechanical stimuli from the microenvironment [13]. YAP, as one of the crucial downstream effectors of this pathway, is involved in various cell physiological processes and homeostatic regulations [14, 15]. The hippo pathway consists of a series of kinases that control the sub-cellular localization and stability of YAP [12]. The mammalian sterile 20-like protein kinase 1 or 2 (MST1/2) and the large tumor suppressor kinase 1 or 2 (LATS1/2) comprise the key to this pathway that, together with the adaptor proteins salvador homolog 1 (SAV1) and Mps one binder kinase activator 1 (MOB1), function in a sequential manner to phosphorylate and inhibit the transcription factors YAP/TAZ [16]. Specifically, the upstream molecules SAV1 and MST1/2 form a complex that phosphorylates and activates the following downstream molecules. MST1/2 kinase can directly phosphorylate and activate LATS1/2 kinase, but binding of the coactivator MOB1 to LATS1/2 also allows autophosphorylation, which in turn phosphorylates YAP/TAZ to promote cytoplasmic sequestration and degradation through the proteasomal pathway [17]. In response to mechanical stimuli, YAP/TAZ is dephosphorylated and translocated to the nucleus. In the absence of DNA-binding domains, the activation of nuclear YAP/TAZ depends on binding to the transcriptional enhancer factor domain family member 1–4 (TEAD1-4), which initiates target gene transcription and expression.

It is also uncertain how mechanical inputs are converted into biochemical and cellular outputs that change gene expression signatures, initiate alveolar bone remodeling, and finally result in tooth movement. Therefore, this study aims to elucidate the relationship between mechanotransduction and mechanical force-induced alveolar bone remodeling during OTM. Specifically, our objectives are: (a) to assess the impact of different force magnitudes on OTM distance, YAP activation, and osteoclastogenesis; (b) to investigate whether the mechanosensor YAP regulates OC formation, thereby initiating alveolar bone remodeling in OTM; and (c) to identify the underlying mechanisms involved in this process. Through these investigations, we aim to provide deeper insights into the cellular and molecular mechanisms governing alveolar bone remodeling during OTM, contributing to therapeutic interventions for improved orthodontic treatment outcomes.

## Results

### Different force magnitudes affect the distance of OTM

**Fig. 1:**
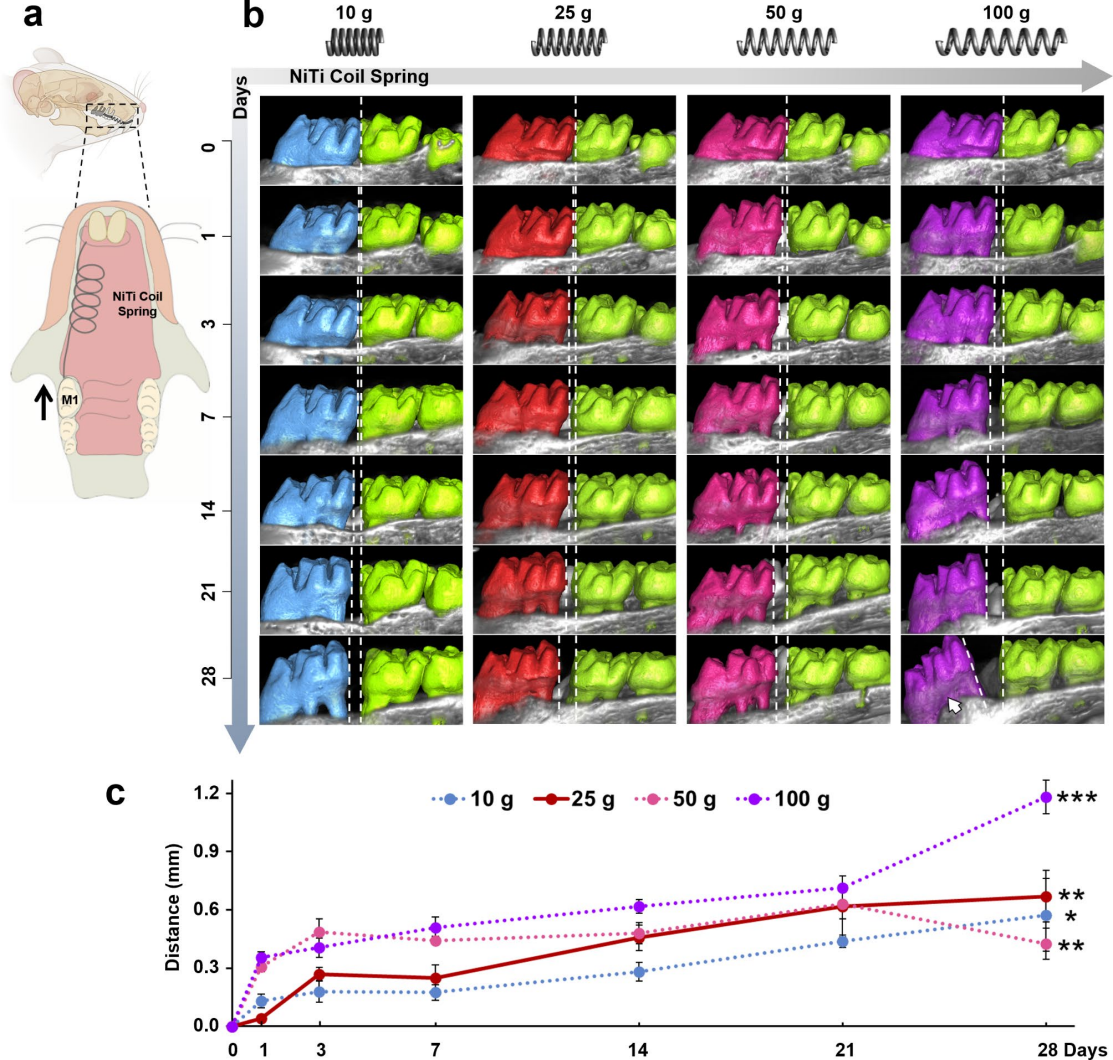
The impact of different force magnitudes on OTM distance. (**a**) Schematic diagram of the rat tooth movement model. The mechanical force was generated by a Ni-Ti coil spring and applied on the right first molar. (**b**) From day 0 to 28 of force application, µCT 3D reconstruction of the maxillary bone and molars in this region depicting changes in tooth movement distance over time for the four force magnitude groups (10, 25, 50, 100 g). (**c**) Quantitative analysis by µCT indicated changes in the distance from the distal crown surface of the first molar to the mesial crown surface of the second molar from day 0 to 28. Each value represents the mean (*n* = 3/group) ± the standard error. * *p* < 0.05, ** *p* < 0.01, *** *p* < 0.001

The effects of different force magnitudes (10, 25, 50, 100 g) on OTM distance are shown in Fig. 1 from day 0 to 28. The µCT-based three-dimensional (3D) reconstructions of maxillae and molars demonstrate that despite different magnitudes in force, all groups exhibit increased OTM distance between the first and second molars over time (Fig. 1b and c). By day 28, significant increases in OTM distance are observed in all groups compared to day 0 (10 g: *p* < 0.05; 25 g: *p* < 0.01; 50 g: *p* < 0.01; 100 g: *p* < 0.001) (Fig. 1c). Morphological and quantitative analysis reveals pronounced mesial tipping of first molars in the 100 g group, contrasting with relatively upright positioning in other groups (Fig. 1b). Notably, the 25 g group shows the second-highest OTM distance, surpassing the 10 g and 50 g groups, without mesial tipping. Additionally, all groups exhibit slight OTM distances on the first day of force application.

### Effects of different force magnitudes on YAP activation and osteoclastogenesis at day 1

**Fig. 2:**
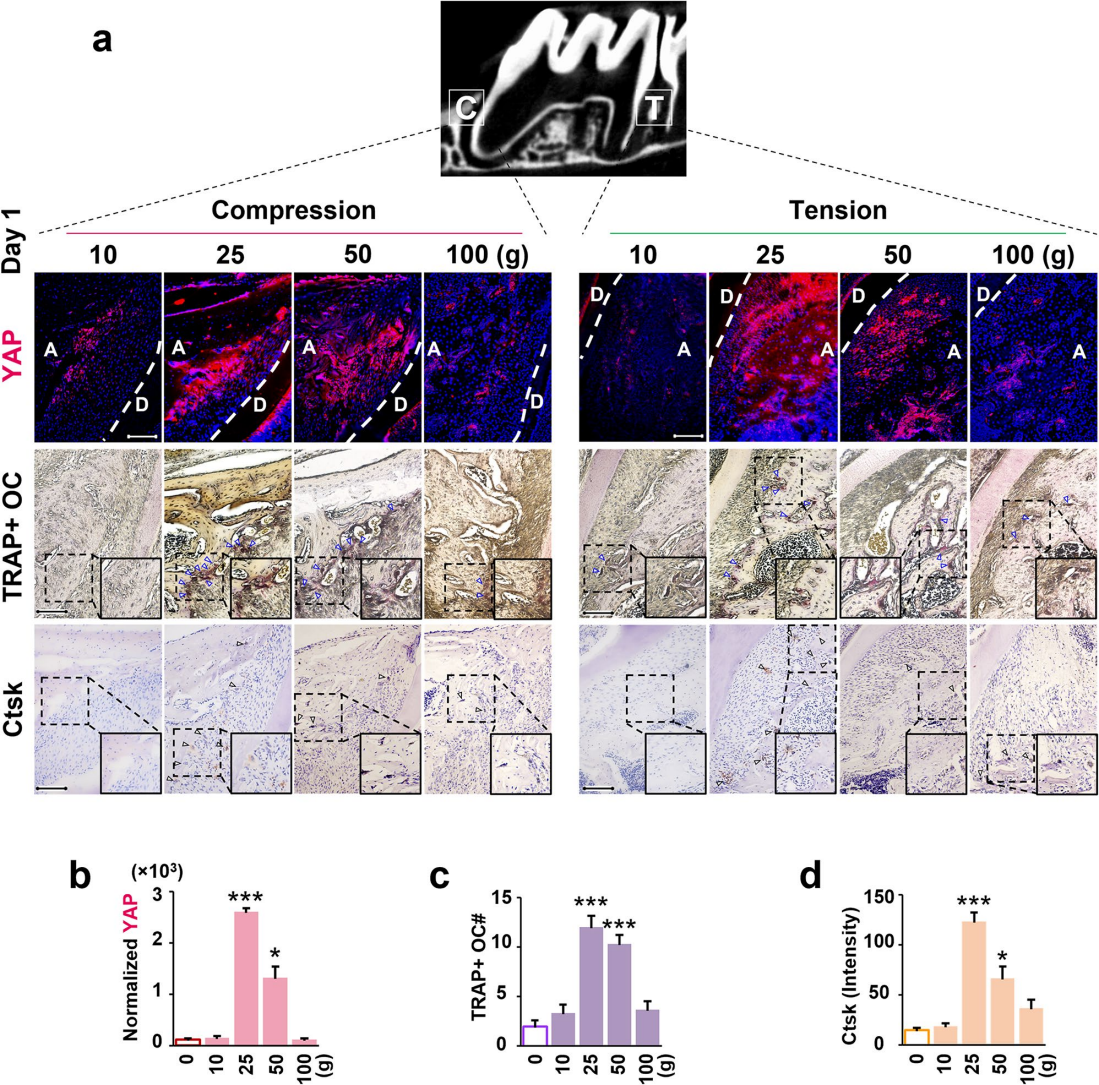
Effects of different force magnitudes on YAP activation and osteoclastogenesis at day 1. (**a**) On the first day of force application, histological staining was used to compare the expression levels of YAP (IF), the number of OCs (TRAP), and the expression level of Ctsk (IHC) in the alveolar bone on the compressive and tensile sides of the first molars in the four force magnitude groups. (**b**) Quantitative analysis of the average expression level of YAP in the alveolar bone under different force magnitudes. (**c**, **d**) Quantitative analysis of the number of OCs and the average expression level of Ctsk under different force magnitudes. Each value represents the mean (*n* = 5/group) ± the standard error. * *p* < 0.05, *** *p* < 0.001. The images were shown with 20× magnification, and high magnification (40×) for indicating TRAP^+^ OCs and the Ctsk expressions

To elucidate the early impact of varying force magnitudes on YAP activation and initial osteoclastogenesis during OTM, we examined histological changes on the first day of force application (Fig. 2). YAP expression was assessed at both compressive and tensile sites of the first molar across four force magnitude groups (Fig. 2a). On day 1, immunofluorescence (IF) staining revealed notably higher YAP activation in the 25 g group compared to others. Quantitative analysis of IF staining (Fig. 2b) showed significant YAP expression on both compressive and tensile sides in the 25 g group, with comparable levels and no significant difference between them (*p* > 0.05). However, YAP expression peaked compared to the 0 g group (*p* < 0.001) and was significantly higher than the 10 g, 50 g, and 100 g groups (*p* < 0.001; *p* < 0.05; *p* < 0.001). YAP expression in the 50 g group was also significantly elevated compared to the 0 g group (*p* < 0.05). Conversely, there was no significant difference in YAP expression between the 10 g and 100 g groups compared to the 0 g group (*p* > 0.05). Supplementary IF staining images of YAP expression for the 0 g group (control) on day 1 are provided in Figure S1.

On the first day of force application, TRAP staining revealed OC formation and recruitment on both compressive and tensile sides (Fig. 2a and c). In the 25 g group, the number of TRAP^+^ OCs significantly increased compared to the 0 g group (*p* < 0.001) and was also higher than the 10 g and 100 g groups (*p* < 0.001; *p* < 0.001). Similarly, in the 50 g group, TRAP^+^ OCs significantly increased compared to the 0 g, 10 g, and 100 g groups (*p* < 0.001; *p* < 0.001; *p* < 0.001). However, there was no significant increase in TRAP^+^ OCs in the 10 g and 100 g groups compared to the 0 g group (*p* > 0.05). Immunohistochemical (IHC) analysis of Ctsk expression, indicating OC activity, followed a similar trend to TRAP^+^ OC quantity and YAP expression (Fig. 2a and d). In the 25 g group, Ctsk expression was enhanced on both compressive and tensile sides, with no significant difference between them (*p* > 0.05). The average Ctsk expression level peaked compared to the 0 g group (*p* < 0.001) and was significantly higher than the 10 g, 50 g, and 100 g groups (*p* < 0.001; *p* < 0.05; *p* < 0.001). Moreover, Ctsk expression in the 50 g group was significantly higher than the 0 g group (*p* < 0.05). However, there was no significant difference in Ctsk expression between the 10 g and 100 g groups compared to the 0 g group (*p* > 0.05). Supplementary TRAP and IHC staining images for the 0 g group (control) on day 1 are provided in Figure S1.

### The variation of YAP expression over time under mechanical force during OTM

**Fig. 3:**
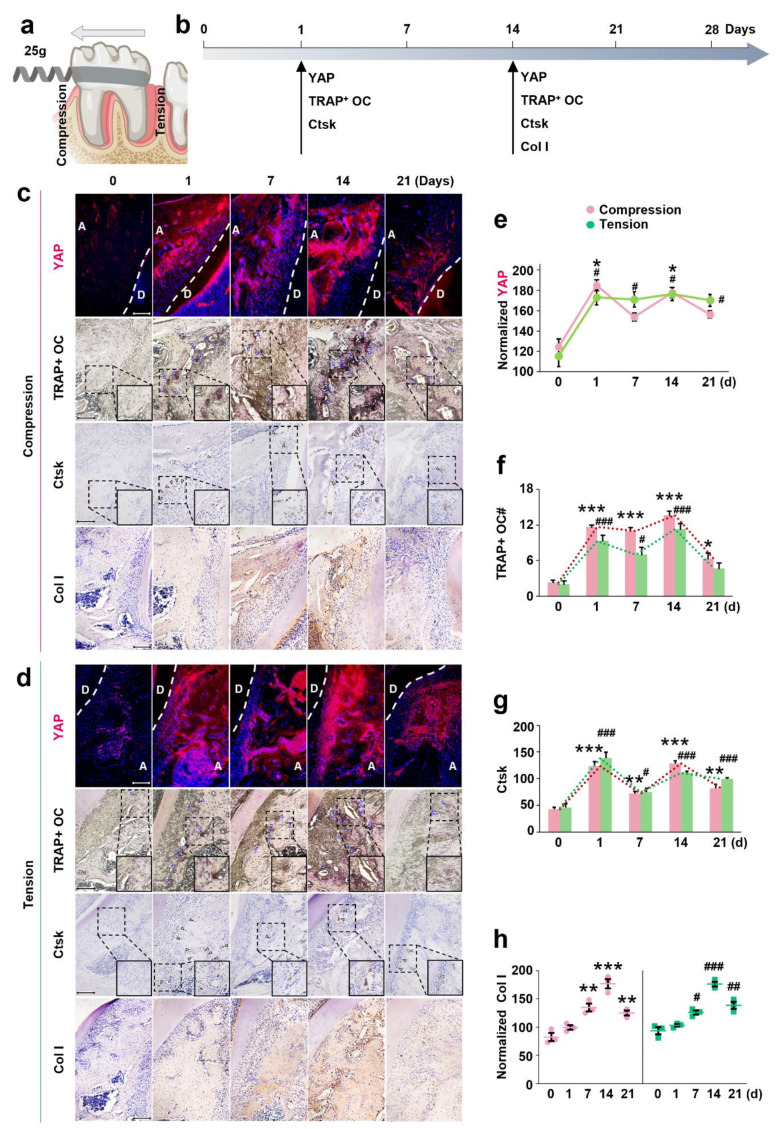
The variation of YAP expression, osteoclastogenesis, and Col I expression over time under mechanical force during OTM. (**a**) Schematic diagram of the rat OTM model using 25 g force magnitude). (**b**) Overall trend of changes over time in the expression of YAP, quantity of TRAP^+^ OCs, and expressions of Ctsk and Col I in the alveolar bone under mechanical force (both compression/tension sides) from day 0 to day 28. (**c**, **d**) Histological changes over time in the expression of YAP (IF), the quantity of TRAP^+^ OCs, and expressions of Ctsk and Col I (IHC) in the alveolar bone under mechanical force (compression/tension sides) from day 0 to day 21. (**e**, **f**, **g**, **h**) Quantitative analysis of changes over time in the expression of YAP (IF), quantity of TRAP^+^ OCs, and expressions of Ctsk and Col I in the alveolar bone under mechanical force (compression/tension) from day 0 to day 21. Each value represents the mean (*n* = 5/group) ± the standard error. Compression side: * *p* < 0.05, ** *p* < 0.01, *** *p* < 0.001. Tension side: ^#^*p* < 0.05, ^##^*p* < 0.01, ^###^*p* < 0.001. The images were shown with 20× magnification, and high magnification (40×) for indicating TRAP^+^ OCs and the Ctsk expressions

From day 0 to day 28, Fig. 3b summarizes the overall trend in YAP expression levels in the alveolar bone under a 25 g mechanical force of OTM. Peaks in YAP response occurred on days 1 and 14. On these days, YAP expression was evident on both compression and tension sides (Fig. 3c and d) with no significant difference between them (*p* > 0.05). Quantitative analysis (Fig. 3e) indicated that YAP levels on the compression side were significantly higher on days 1 and 14 compared to day 0 (*p* < 0.05; *p* < 0.05). On days 7 and 21, YAP levels on the compression side decreased but remained above day 0 levels. On the tension side, YAP levels remained relatively high from day 1 to day 21, significantly higher than on day 0 (*p* < 0.05 for all). Figure S1 includes IF staining images showing changes in YAP expression in the alveolar bone over time under mechanical force from day 0 to day 28.

### The variation of osteoclastogenesis over time under mechanical force during OTM

During days 0–28, Fig. 3b summarizes the overall trends in TRAP^+^ OC quantity and Ctsk expression in the alveolar bone under a 25 g mechanical force of OTM. Peaks in TRAP^+^ OC quantity and Ctsk expression occurred on days 1 and 14, respectively. On these days, both markers significantly increased on the compression and tension sides (Fig. 3c and d), with no significant difference between them (*p* > 0.05). Quantitative analysis (Fig. 3f) showed that TRAP^+^ OCs remained high on the compression side from days 1 to 21 (*p* < 0.001; *p* < 0.001; *p* < 0.001; *p* < 0.05). On the tension side, the number of TRAP^+^ OCs was more on days 1 and 14 (*p* < 0.001; *p* < 0.001), decreased by day 7 but remained elevated compared to day 0 (*p* < 0.05), and showed no significant difference by day 21 (*p* > 0.05). Similarly, Ctsk expression was high on both sides on days 1 and 14. Quantitative analysis (Fig. 3g) indicated a significant increase in Ctsk expression on the compression side from days 1 to 21 (*p* < 0.001; *p* < 0.01; *p* < 0.001; *p* < 0.01) and a similar trend on the tension side (*p* < 0.001; *p* < 0.05; *p* < 0.001; *p* < 0.001). Changes in TRAP^+^ OCs and Ctsk expression under mechanical force from days 0 to 28 are depicted in Figure S1.

### The variation of type I collagen over time under mechanical force during OTM

To preliminarily explore the sequential remodeling pattern of bone resorption and formation in response to mechanical stimuli, we investigated the changes in type I collagen (Col I) expression, a marker of osteoblast activity, over time. Figure 3b shows the trend in Col I expression in the alveolar bone under a 25 g mechanical force from days 0 to 28 of OTM, with a peak on day 14. From days 7 to 21, Col I was significantly expressed on both compression and tension sides (Fig. 3c and d), with no significant difference between them (*p* > 0.05). Quantitative analysis (Fig. 3h) indicated that on days 7, 14, and 21, Col I expression on the compression side peaked, significantly higher than on day 0 (*p* < 0.01; *p* < 0.001; *p* < 0.01). Similarly, on the tension side, Col I expression peaked on day 14 (*p* < 0.001) and remained significantly higher on days 7 and 21 compared to day 0 (*p* < 0.05; *p* < 0.01). Changes in Col I expression under mechanical force from days 0 to 28 are illustrated in Figure S1.

### The KEGG pathway enrichment analysis of alveolar bone under mechanical force in the early stages

**Fig. 4:**
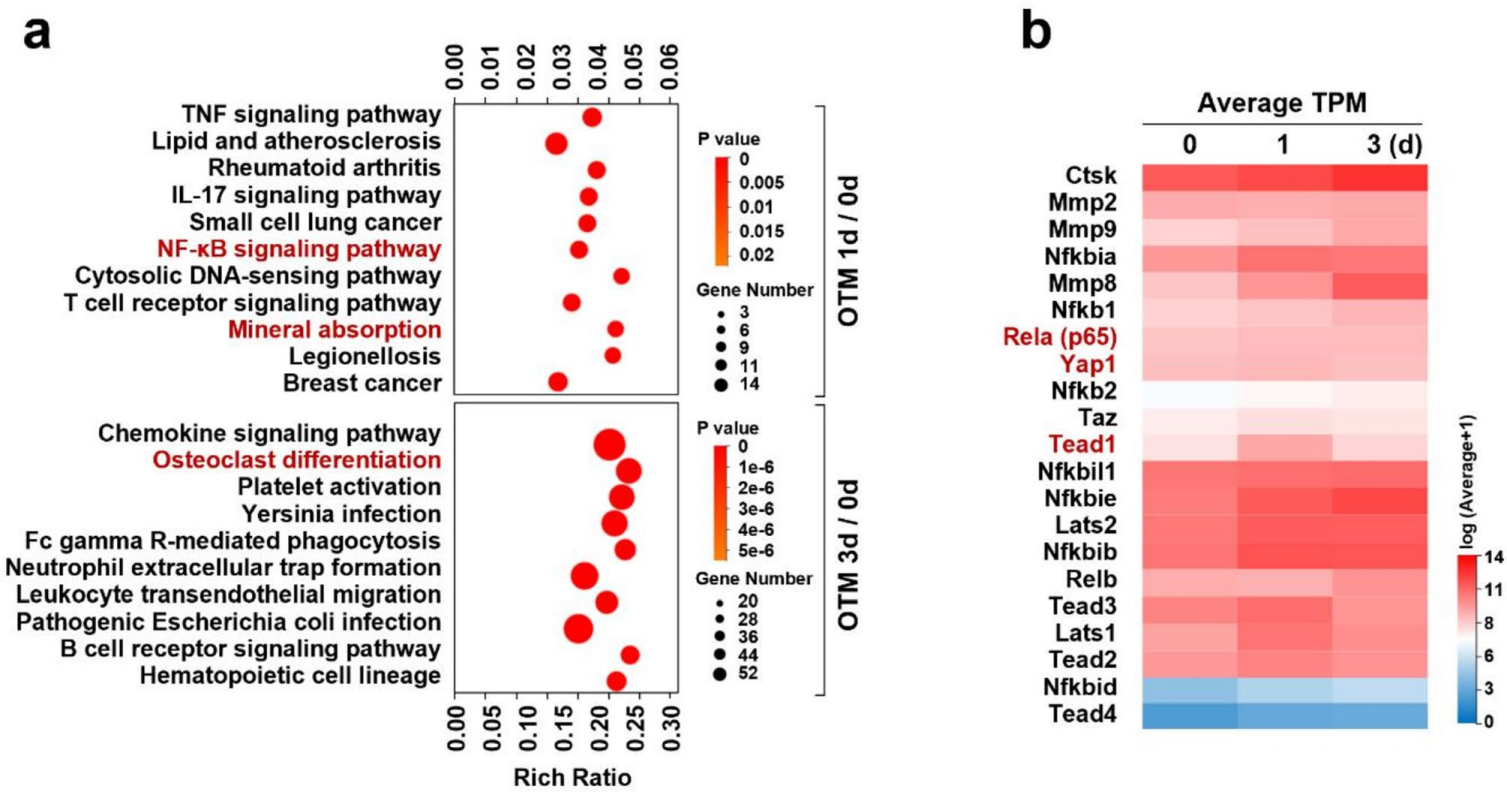
The RNA-Seq analysis of alveolar bone under mechanical force in the early stages. (**a**) The KEGG pathway enrichment analysis of alveolar bone under mechanical force in the early stages (1–3 days). (**b**) Cluster heatmap of differential genes expression analysis on alveolar bone under mechanical force in the early stages (1–3 days). Each value represents the mean (*n* = 4/group) ± the standard error

Using RNA-sequencing (RNA-seq) technology, we analyzed KEGG pathway enrichment in the early stages (1–3 days) of alveolar bone remodeling induced by mechanical force during OTM. Compared to the 0-day group, significant increases were observed in the top 10 enriched pathways on day 1 of OTM, including the nuclear factor kappa B (NF-κB) signaling pathway and pathways related to mineral absorption (*p* < 0.01; *p* < 0.01) (Fig. 4a). The NF-κB signaling pathway is crucial for osteoclast formation and differentiation. Activation of mineral absorption pathways in the early stages (1 day) of alveolar bone remodeling indicates upregulation of enzymes, including Ctsk, matrix metalloproteinase-8 and 9 (MMP-8, -9), and inflammation-related pathways. Indeed, the top 10 enriched pathways also included TNF and IL-17 signaling pathways and other inflammatory pathways, confirming that in the initial stages of mechanical force-induced alveolar bone remodeling, the earliest activation occurs in pathways associated with promoting OC differentiation and bone resorption on both compression and tension sides. Further analysis (Fig. 4a) on day 3 of OTM compared to the 0-day group revealed more enriched pathways, with significant increases observed in pathways related to OC differentiation (*p* < 0.001) and the chemokine signaling pathway, closely linked to inflammation. These findings clarify that in the initial stages of mechanical force-induced alveolar bone remodeling, the earliest activation occurs in inflammatory pathways promoting OC differentiation and bone resorption, consistent with previous findings [11]. Additionally, KEGG-enriched pathways in the initial stages (1–3 days) did not show significant activation of pathways associated with osteogenesis at this early time.

### Cluster heatmap of differential genes expression analysis on alveolar bone under mechanical force in the early stages

Through RNA-Seq technology, a clustering heatmap of differentially expressed target genes in the early stages (1 and 3 days) of alveolar bone remodeling under mechanical force was generated. Figure 4b illustrates YAP1, RelA (NF-κB p65) exhibited significantly higher expression levels on OTM day 1 compared to day 0 and OTM day 3. Additionally, other upstream and downstream factors of the Hippo signaling pathway, such as LATS1/2, TAZ, and TEAD1/2/3, showed significantly higher expression levels on OTM day 1 compared to day 0 and OTM day 3. Furthermore, the analysis also revealed that the expression levels of osteoclastic markers, including Ctsk, MMP-8, and MMP-9 significantly increased on OTM day 1 compared to day 0, with further significant increases observed on OTM day 3.

### Effects of compression on YAP expression and osteoclast differentiation in rat osteoclasts (ex-vivo)

**Fig. 5:**
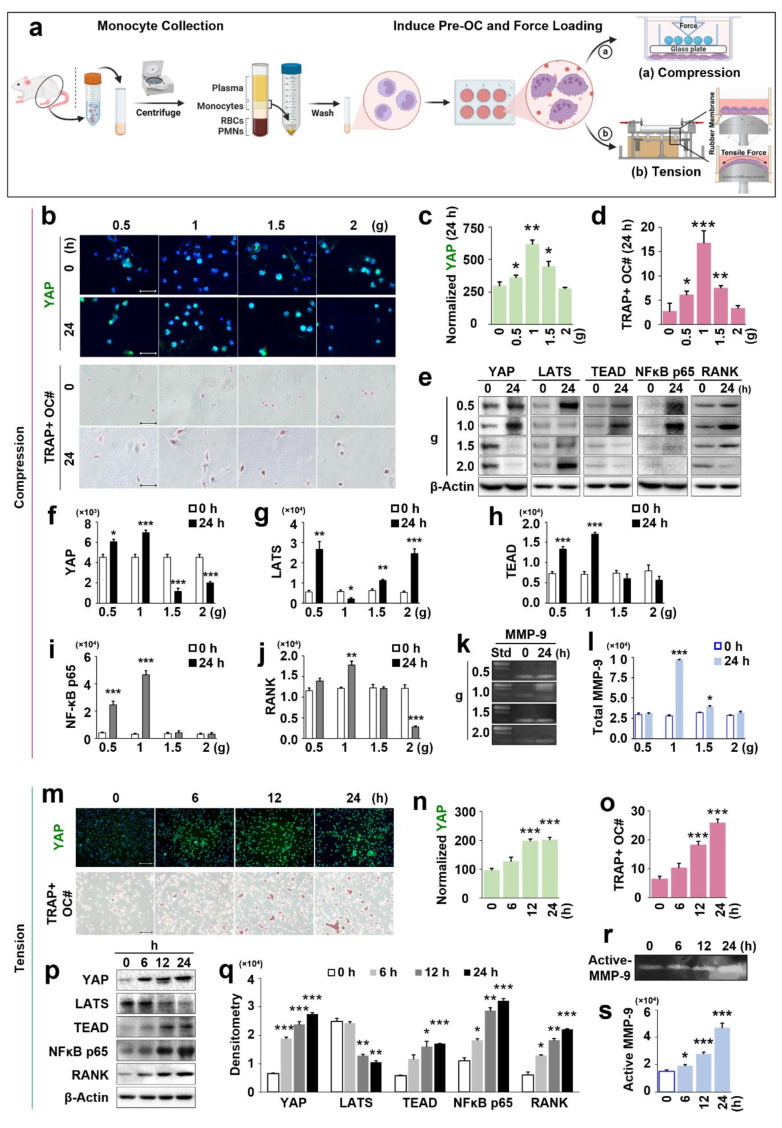
Effects of compression/tension on YAP expression and osteoclastogenesis via YAP/TEAD/NF-κB p65 signaling pathway in rat osteoclasts (ex-vivo). (**a**) Schematic diagram of establishing an ex-vivo cell culture model for stimulating primary rat OC precursors (monocytes) with mechanical force (compression/tension). (**b**) Effects of compression at different magnitudes on the expression of YAP in rat OCs (IF) and the differentiation of TRAP^+^ OCs. (**c**, **d**) Quantitative analysis of the expression of YAP in rat OCs and the number of TRAP^+^ OCs under different magnitudes of compression. (**e**) WB detection of the expression of YAP, LATS, TEAD, NF-κB p65, and RANK in rat OCs under different magnitudes of compression. (**f**-**j**) Quantitative analysis of the expression of YAP, LATS, TEAD, NF-κB p65, and RANK in rat OCs under different magnitudes of compression. (**k**, **l**) Gelatin zymography to detect the effects of different magnitudes of compression on the expression of MMP-9 in rat OCs, as well as quantitative analysis. (**m**) Effects of tension on the expression of YAP in rat OCs (IF) and the differentiation of TRAP^+^ OCs. (**n**, **o**) Quantitative analysis of the expression of YAP in rat OCs and the number of TRAP^+^ OCs over time under tension. (**p**) WB detection of the changes in the expression of YAP, LATS, TEAD, NF-κB p65, and RANK in rat OCs over time under tension. (**q**) Quantitative analysis of the changes in the expression of YAP, LATS, TEAD, NF-κB p65, and RANK in rat OCs over time under tension. (**r**, **s**) Gelatin zymography to detect the effects of tension overtime on the expression of MMP-9 in rat OCs, as well as quantitative analysis. Each value represents the mean (*n* = 4/group) ± the standard error. * *p* < 0.05, ** *p* < 0.01, *** *p* < 0.001. The images were shown with 40× magnification

The changes in YAP expression and OC differentiation in rat OCs (ex-vivo) under different compression magnitudes (0.5, 1, 1.5, 2 g) are depicted in Fig. 5b. At 24 h, the expression of YAP significantly increased under compression magnitudes of 0.5, 1, and 1.5 g compared to the 0 g control group (*p* < 0.05; *p* < 0.01; *p* < 0.05) (Fig. 5c). Among these, the 1 g group exhibited the highest expression of YAP. However, at 24 h, there was no increase in YAP expression in the 2 g group. The changes in YAP expression over time (6 and 12 h) in rat OCs under different compression magnitudes (0.5, 1, 1.5, 2 g) are depicted in Figure S2.

Similarly, the number of TRAP^+^ OCs significantly increased under compression magnitudes of 0.5, 1, and 1.5 g compared to the 0 g control group (*p* < 0.05; *p* < 0.001; *p* < 0.01) (Fig. 5d). Likewise, the 1 g group exhibited the highest number of TRAP^+^ OCs. Maturely differentiated OCs, characterized by multinucleated cells with TRAP^+^ staining, were observed (Fig. 5b). However, at 24 h, there was no increase in the number of TRAP^+^ OCs in the 2 g group, with only a small amount of OC differentiation observed.

### Effects of compression on YAP/TEAD/NF-κB p65 signaling pathway in mediating osteoclast differentiation (ex-vivo) in the early stage

The WB results (Fig. 5e) showed that at 24 h, the expression of YAP in OCs significantly increased under compression magnitudes of 0.5 and 1 g compared to the control group (*p* < 0.05; *p* < 0.001) (Fig. 5f). Among these, the 1 g group exhibited the highest expression of YAP. However, the expression of YAP in OCs significantly decreased in the 1.5 and 2 g groups compared to the control group (*p* < 0.001; *p* < 0.001).

Further WB analysis revealed that different compression magnitudes had an impact on LATS and TEAD which are two factors in the Hippo pathway (Fig. 5e). At 24 h, under 1 g of compression, the expression of LATS in OCs significantly decreased compared to the control group (*p* < 0.05) (Fig. 5g). However, in the other three groups (0.5, 1.5, 2 g), the expression of LATS in OCs significantly increased compared to the control group (*p* < 0.01; *p* < 0.01; *p* < 0.001).

Additionally, at 24 h, under 0.5 and 1 g of compression, the expression of TEAD in OCs significantly increased compared to the control group (*p* < 0.001; *p* < 0.001). Among these, the 1 g group exhibited the highest expression of TEAD (Fig. 5h). However, there was no significant difference in the expression of TEAD in OCs in the 1.5 and 2 g groups compared to the control group (*p* > 0.05; *p* > 0.05).

The analysis also found that different compression levels affected NF-κB p65 and RANK which are two important factors in the NF-κB pathway (Fig. 5e). At 24 h, under 0.5 and 1 g of compression, the expression of NF-κB p65 in OCs significantly increased compared to the control group (*p* < 0.001; *p* < 0.001) (Fig. 5i). Among these, the 1 g group exhibited the highest expression of NF-κB p65. However, there was no significant difference in the expression of NF-κB p65 in OCs in the 1.5 and 2 g groups compared to the control group (*p* > 0.05; *p* > 0.05). On the other hand, at 24 h, under 1 g of compression, the expression of RANK in OCs significantly increased compared to the control group (*p* < 0.01) (Fig. 5j). However, there was no significant difference in the expression of RANK in OCs in the 0.5 and 1.5 g groups compared to the control group (*p* > 0.05; *p* > 0.05). Meanwhile, the expression of RANK in OCs significantly decreased in the 2 g group compared to the control group (*p* < 0.001).

### Effect of compression on MMP-9 expression in rat osteoclasts (ex-vivo)

Gelatin zymography experiments revealed (Fig. 5k) that at 24 h, under compression magnitudes of 1 and 1.5 g, the total MMP-9 expression in OCs significantly increased compared to the control group (*p* < 0.001; *p* < 0.05). Among them, the total MMP-9 expression in OCs was highest in the 1 g group (Fig. 5l). However, there was no significant difference in total MMP-9 expression in OCs between the 0.5 g and 2 g groups compared to the control group (*p* > 0.05; *p* > 0.05).

### Effects of tension on YAP expression and osteoclast differentiation in rat osteoclasts (ex-vivo)

The changes in YAP expression and OC differentiation in rat OCs (ex-vivo) under tension (0.5, 1, 1.5, 2 g) over time are depicted in Fig. 5m. Under tension from 0 to 24 h, the expression of YAP in rat OCs gradually increased over time (Fig. 5m). Specifically, at 12 and 24 h of loading tension, the expression of YAP significantly increased compared to 0 h (*p* < 0.001; *p* < 0.001). Moreover, the expression of YAP in OCs reached its peak at 24 h (Fig. 5n).

Similarly, under tension from 0 to 24 h, the number of TRAP^+^ OCs in rats gradually increased over time (Fig. 5m). Again, maturely differentiated OCs, characterized by multinucleated cells with TRAP^+^ staining, were observed. At 12 and 24 h of loading tension, the number of TRAP^+^ OCs significantly increased compared to 0 h (*p* < 0.001; *p* < 0.001) (Fig. 5o). Additionally, the group at 24 h had the highest number of TRAP^+^ OCs. However, there was only a small amount of OC differentiation observed at 6 h, with no significant difference compared to 0 h (*p* > 0.05).

### Effects of tension on YAP/TEAD/NF-κB p65 signaling pathway in mediating osteoclast differentiation (ex-vivo)

The WB results (Fig. 5p) showed that under tension for 6, 12, and 24 h, the expression of YAP in OCs gradually increased over time, with significant differences compared to the 0-hour control group (*p* < 0.001; *p* < 0.001; *p* < 0.001). Among these, the group at 24 h exhibited the highest expression of YAP (Fig. 5q).

Further analysis revealed that tension also affected two factors in the Hippo pathway (Fig. 5p and q). Under tension from 0 to 24 h, the expression of LATS in OCs gradually decreased over time. Compared to the 0-hour control group, the expression levels of this protein at 12 and 24 h showed significant differences (*p* < 0.01; *p* < 0.01) (Fig. 5q). Among these, the group at 24 h had the lowest expression of LATS. Additionally, under tension from 0 to 24 h, the expression of TEAD protein increased gradually over time. The expression levels of this protein showed significant differences at 12 and 24 h (*p* < 0.05; *p* < 0.001), compared to the 0-hour control group (Fig. 5q).

Quantitative analysis also revealed that tension affected two factors of the NF-κB pathway (Fig. 5p and q). Under tension for 6, 12, and 24 h, the expression of NF-κB p65 in OCs increased gradually over time, with significant differences compared to the 0-hour control group (*p* < 0.05; *p* < 0.01; *p* < 0.001). Among these, the group at 24 h exhibited the highest expression of NF-κB p65 (Fig. 5q). Furthermore, under tension for 6, 12, and 24 h, the expression of RANK showed a similar trend to NF-κB p65, increasing gradually over time, with significant differences compared to the 0-hour control group (*p* < 0.05; *p* < 0.01; *p* < 0.001). Among these, the group at 24 h exhibited the highest expression of RANK (Fig. 5q).

### Effect of tension on MMP-9 expression in rat osteoclasts (ex-vivo)

Through gelatin zymography experiments (Fig. 5r), it was found that under tension for 6, 12, and 24 h, the expression of total MMP-9 in OCs gradually increased over time, with significant differences compared to the 0-hour control group (*p* < 0.05; *p* < 0.001; *p* < 0.001). Among these, the group at 24 h exhibited the highest expression of total MMP-9 (Fig. 5s).

### YAP inhibition affects OTM distance under mechanical force (in vivo)

**Fig. 6:**
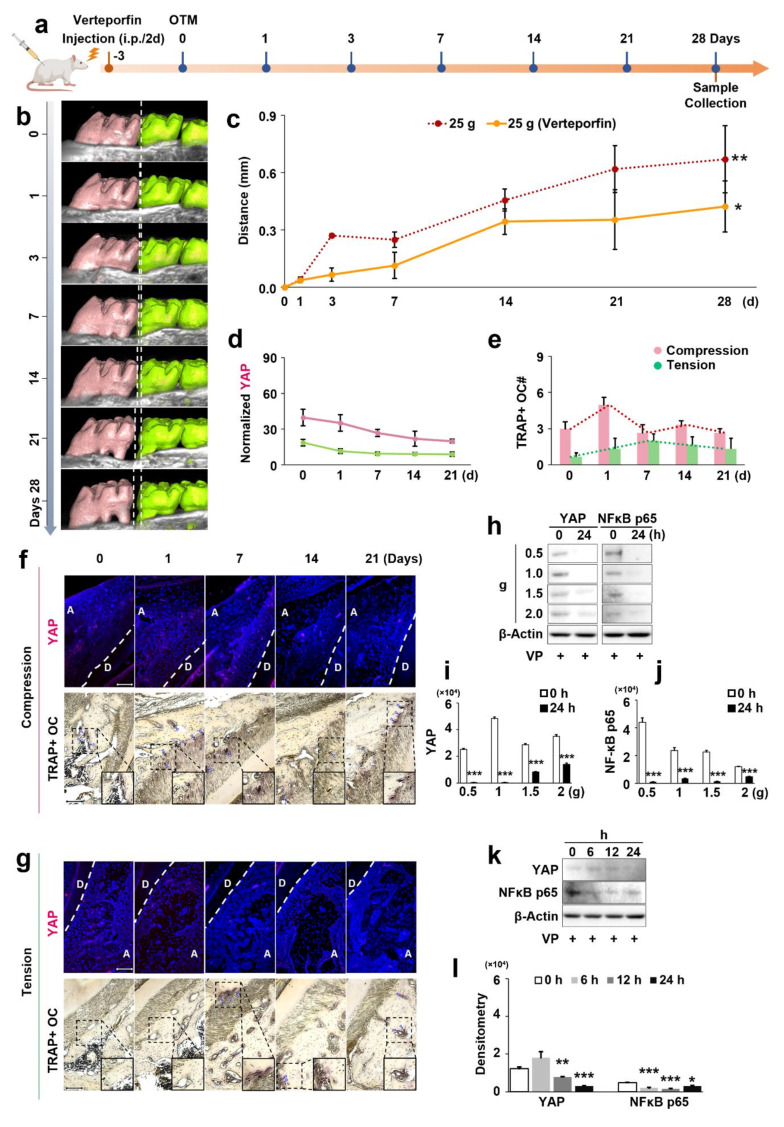
Effects of inhibiting YAP on OTM distance and osteoclastogenesis in alveolar bone remodeling under mechanical force (in vivo), and on the expression of NF-κB p65 in rat osteoclasts (ex-vivo). (**a**) Establishment of the rat’s OTM model with in vivo YAP inhibition. (**b**) µCT 3D reconstruction of the maxilla and tooth movement region from day 0 to 28 under mechanical force. (**c**) Effects of in vivo YAP inhibition on OTM distance under mechanical force and quantitative analysis. (**d**-**g**) Effects of in vivo YAP inhibition on YAP expression and TRAP^+^ OC quantity within the alveolar bone, along with quantitative analysis. (**h**-**l**) Effects of inhibiting YAP in OCs on NF-κB p65 expression under mechanical force (compression/tension), along with quantitative analysis. For the in vivo study, each value represents the mean (*n* = 3/group) ± the standard error. For the ex-vivo study, each value represents the mean (*n* = 4/group) ± the standard error. * *p* < 0.05, ** *p* < 0.01, *** *p* < 0.001. The images were shown with 20× magnification, and high magnification (40×) for indicating TRAP + OCs

The in vivo effects of YAP inhibition with verteporfin (VP group) on OTM distance under mechanical force (25 g) were assessed through intraperitoneal injection of VP to suppress YAP expression from day 0 to 28 (Fig. 6a). As depicted in Fig. 6b, at day 28, 3D reconstruction of rats’ maxillae and molars in the VP group using µCT showed a slight increase in OTM distance over time under mechanical stimuli. However, YAP inhibition significantly slowed down the OTM process. Notably, the VP group exhibited a shorter OTM distance between the first and second molars compared to the normal 25 g group without YAP inhibition (*p* < 0.05) (Fig. 6c).

### The impact of YAP inhibition on osteoclastogenesis in mechanical force-induced alveolar bone remodeling during OTM

The IF staining of alveolar bone tissue showed that from day 0 to 21 of force application during OTM, YAP expression was largely inhibited on the compression and tension sides (Fig. 6f and g), with similar expression levels on both sides and no significant difference between them (*p* > 0.05) (Fig. 6d). This reflects the effective inhibitory action of VP on YAP. The IF staining images of YAP inhibition in the alveolar bone over time (from day 0 to 28) with VP injection under mechanical force are presented in Figure S3.

TRAP staining results revealed that from day 0 to 21 of force application, TRAP^+^ OCs were still visible on the compression and tension sides during OTM, but in lower numbers (Fig. 6f and g). Specifically, quantitative analysis showed that from day 1 to 21, there was no significant difference in the number of TRAP^+^ OCs on the compression side compared to day 0 (*p* > 0.05) (Fig. 6e). Similarly, the number of TRAP^+^ OCs on the tension side remained at a lower level throughout, with no significant difference compared to day 0 (*p* > 0.05). The images of TRAP staining in the alveolar bone over time (from day 0 to 28) with VP injection under mechanical force (compression/tension) are presented in Figure S3.

### The influence of inhibiting YAP on NF-κB p65 expression in rats’ OCs (ex-vivo) under mechanical force (Compression/Tension)

On the one hand, WB results (Fig. 6h) showed that at 24 h, the expression of YAP in OCs under different compression magnitudes (0.5, 1, 1.5, and 2 g) was significantly reduced compared to the control group (*p* < 0.001; *p* < 0.001; *p* < 0.001; *p* < 0.001) (Fig. 6i), indicating significant inhibition of YAP by VP. Importantly, at 24 h, the expression of NF-κB p65 was also significantly decreased compared to the control group (*p* < 0.001; *p* < 0.001; *p* < 0.001; *p* < 0.001) (Fig. 6j). This result suggests that under mechanical force, YAP may regulate the differentiation of OCs via NF-κB p65.

On the other hand, at 12 and 24 h, the expression of YAP in OCs under tension was also significantly reduced compared to the 0-hour control group (*p* < 0.01; *p* < 0.001) (Fig. 6k and l). Importantly, at 6, 12, and 24 h, the expression of NF-κB p65 was also significantly decreased compared to the 0-hour control group (*p* < 0.001; *p* < 0.001; *p* < 0.05) (Fig. 6l). With a trend similar to the results of the compression group, it further clarifies that YAP likely mediates the differentiation of OCs through NF-κB p65.

## Discussion

It is generally believed that during OTM, alveolar bone remodeling involves bone formation on the tension side and bone resorption on the compression side. However, by previous studies on orthodontic force-inducing alveolar bone remodeling [11] and orthopedic force-inducing maxillofacial suture remodeling, [18] we have observed that regardless of the type of force applied (compression/tension), early-stage bone remodeling under mechanical force entails the recruitment of a significant number of OCs and inflammatory cells to the alveolar bone tissue. Conversely, OB-mediated bone formation is notably limited in the early stages, even under tension, leading to an asynchronous remodeling pattern [19] where OC-mediated bone resorption precedes OB-mediated bone formation.

In our study, we observed significant OC recruitment to both compression and tension sides of the alveolar bone during early force application, with no notable differences between them. However, OBs were not observed on either the compression or tension side during the early application of force (1–3 days). Additionally, Col I expression peaked on day 14, suggesting that OB-mediated bone formation occurred subsequent to OC-mediated bone resorption during OTM. Importantly, preceding bone resorption may be a prerequisite for the initiation of subsequent bone formation during OTM. Newly formed bone by OBs replaces precisely the amount removed by OC-mediated bone resorption at the same level, thus promoting tooth movement. Emerging evidence suggests that bone formation is induced by osteoclastic bone resorption or the presence of OCs themselves in bone remodeling [20]. Studies have indicated the involvement of OC-secreted factors in the coupling process [21–24].

To mimic the in vivo study of mechanical force (compression/tension)-induced alveolar bone remodeling during OTM, we also designed the ex-vivo study by using pre-OCs under compression/tension forces. We further explore the underlying mechanisms of how the mechanosensor YAP regulates OC formation, thereby initiating alveolar bone remodeling. In this study, both compressive and tensile forces induced pre-OCs to fully differentiate to mature and functional OCs, detected by increased TRAP^+^ staining with high expressions of total MMP-9.

Interestingly, KEGG pathway enrichment and cluster heatmap analyses revealed active OC differentiation pathways on day 3 of OTM, with significantly higher expression of functional proteins such as Ctsk, MMP-8, and MMP-9 compared to days 1 and 0 of OTM. This suggests that during the initial stages of alveolar bone remodeling under mechanical force during OTM, OC-mediated bone resorption is prominent, prior to OB-mediated bone formation, reinforcing the concept of asynchronous remodeling (a temporal sequence remodeling pattern). Whether this pattern mirrors the physiological bone remodeling process observed in basic multicellular units (BMUs) in long bones will be investigated in our future studies [25].

Furthermore, different force magnitudes impact OTM distance and osteoclastogenesis, with lighter forces (in vivo: 25 g; ex-vivo:1 g) favoring OC differentiation and alveolar bone remodeling. Notably, on day 14, we observed a simultaneous increase in TRAP^+^ OCs, Ctsk expression, and Col I expression, suggesting the initiation of a “second wave” of alveolar bone remodeling coinciding with the “first wave,” enhancing bone resorption. Additionally, Lassen et al., [6] demonstrated the presence of primary OCs at the tip of the bone remodeling tunnel, while those involved in interrupting reversal episodes were identified as secondary OCs. These alternations, involving primary OCs, reversal cells, and secondary OCs, contribute to the eroded surface of bone subjected to these processes.

Regarding the role of YAP in mechanical-induced bone remodeling, it was demonstrated YAP expressions were upregulated 12 h to 14 days after loading force in an experimental OTM study in rats, suggesting that YAP may be involved in the proliferation and differentiation of periodontal cells [15] Importantly, studies suggested that osteoclastogenesis and resorptive activity of bone are regulated by YAP [26–28]. Knockdown of YAP1 in bone marrow-derived macrophages (BMM) downregulated osteoclastogenesis, decreased osteoclastic activities and reduced the formation of F-actin sealing zone in resorptive pits [26]. Besides, disruption of YAP/TEAD association by Verteporfin also impaired the NF-κB signaling pathway [26]. Recently, a growth differentiation factor 15 (GDF15) has been found to play a role in the relation between osteoclastogenesis and YAP. GDF15 is regulated by YAP, which promotes the expressions of proinflammatory cytokines and RANKL/OPG ratio in the functional modulation of OC progenitors in response to a compressive force, providing novel insights into the molecular mechanism of mechanotransduction during OTM [28].

A limitation of this study is that verteporfin (VP), a YAP inhibitor, can affect various cellular processes, including autophagy (without light activation) [29] and cell proliferation. However, VP may also induce tumor cell apoptosis [30] and enhance p53 sensitivity in tumors [31]. To minimize side effects, VP was administered to rats via intraperitoneal injection for only one month in our study [32].

**Fig. 7:**
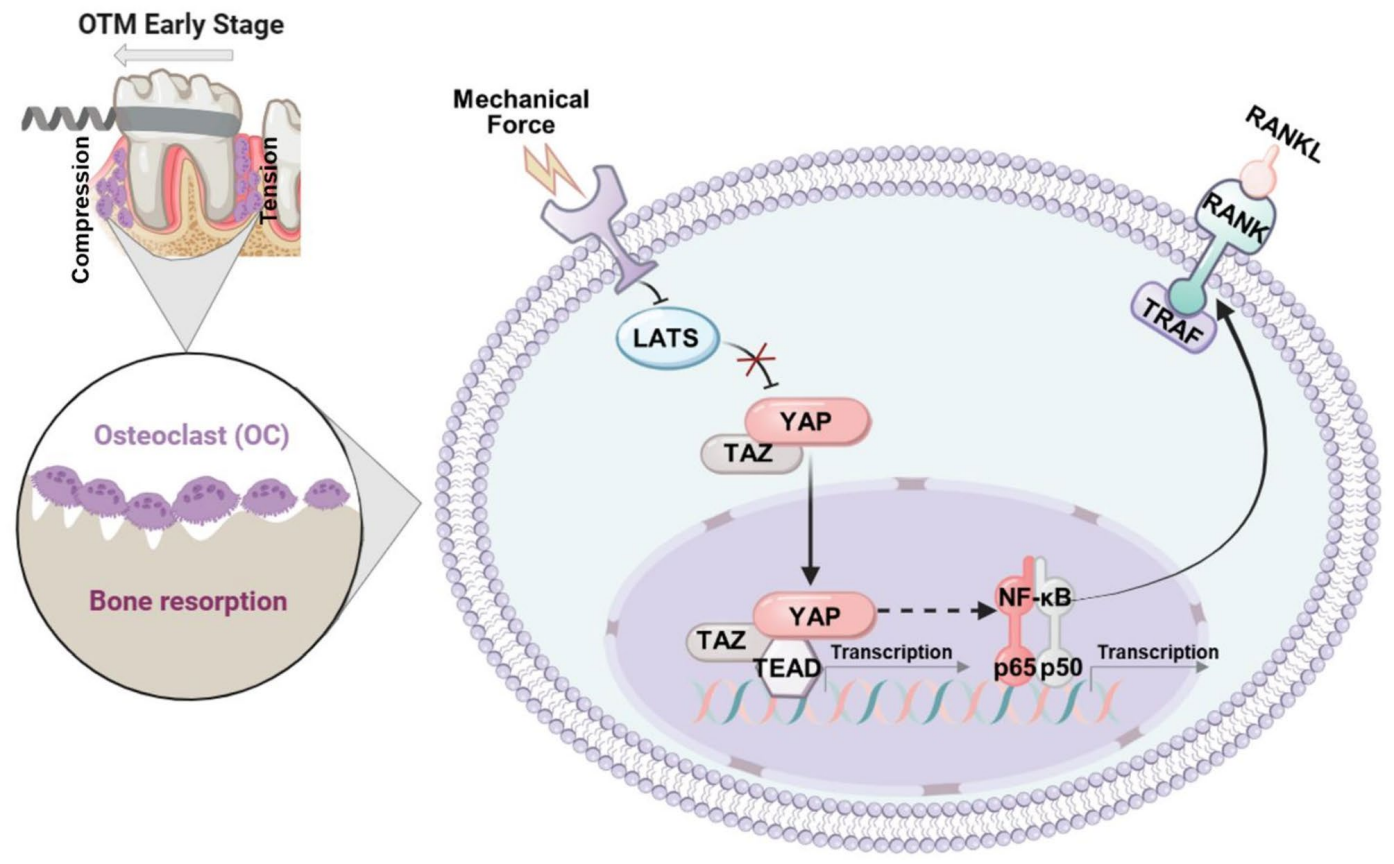
The molecular mechanism elucidated in this study. In the early stage of OTM, the mechanosensor YAP functions through two factors (LATS and TEAD) of the Hippo signaling pathway. While YAP is activated, it may promote OC formation and differentiation by activating NF-κB p65 and RANK, thereby initiating alveolar bone remodeling

Building upon past and current findings, we demonstrated the OC activities at the beginning of bone remodeling during OTM were regulated by a YAP/TEAD/NF-κB p65 signaling pathway. During the early stages of force application, mechanical force (compression/tension) activates YAP, likely through two factors (LATS and TEAD) of the Hippo signaling pathway. This activation is accompanied by a decrease in the upstream LATS and an increase in downstream TEAD expression. Reduced expression of LATS, which mediates a cascade of phosphorylation reactions promoting YAP degradation in the cytoplasm, favors an increase in YAP expression [26, 33]. Notably, increased YAP can translocate to the nucleus, bind to TEAD in the nucleus, and initiate transcription [34]. Additionally, we have found YAP activation is accompanied by an increase in NF-κB p65 expression; and inhibition of YAP also affects NF-κB p65 expression. The latter can promote RANK expression via the NF-κB signaling pathway, thereby facilitating OC formation, differentiation, and maturation. Therefore, subsequent alveolar bone remodeling under mechanical force is initiated by OC-mediated bone resorption. The molecular mechanism elucidated in this study is summarized in Fig. 7.

## Conclusions

In conclusion, our study provides insight into the asynchronous remodeling pattern of alveolar bone during OTM. Both compression and tension influence early remodeling by promoting OC formation, with minimal impact on OBs initially. Force magnitude affects OTM distance, suggesting lighter forces are optimal for alveolar bone remodeling. YAP mediates OC formation via NF-κB p65, initiating bone remodeling and facilitating OTM. These findings offer scientific evidence to orthodontists on optimizing force application for efficient and healthy tooth movement.

## Materials and methods

### Ethics of animal studies

One hundred and thirty male SD rats (weight/age: 50 g/21 days) were purchased from Beijing Vital River (China) and housed at Peking University School and Hospital of Stomatology. Animal care was provided by the center’s personnel. Research protocols for animal studies were approved by the Peking University Biomedical Ethics Committee (LA2020515) and adhered to ARRIVE 2.0 [35] guidelines for ethical animal research. The Three Rs (3Rs) were reviewed by the Committee and approved. All rats were single housed in proper sterile-filter capped cages, and were given unlimited access to food and water. The ambient temperature was maintained at 18–24 °C, and the lights were automatically turned on/off at 06:00 AM/18:00 PM.

### Establishment of a rat model of orthodontic tooth movement (OTM) with different magnitudes of mechanical force

A total of ninety-three rats were randomly allocated into Control (C) and OTM (O) groups. For the O group, different mechanical force magnitudes (10, 25, 50, 100 g) were applied to the maxillary right first molars, while in the C group, no force was applied (0 gram). Specifically, anesthesia was induced in seventy-two OTM rats intraperitoneally (50–60 mg/kg, 1% pentobarbital sodium, i.p.), and the right maxillary first molars were subjected to various force magnitudes to move towards the mesial side. A custom-made orthodontic nickel-titanium coil (0.2 mm wire diameter, 1 mm loop diameter, 6.5 mm length) was used, anchored between the central incisor and first molar with ligature wires (0.2 mm diameter) reinforced by light-curing resin. After the establishment of the OTM rat models (Figs. 1a and 3a), the coil springs were monitored daily without reactivation, and rats with loose springs were excluded. Specimens were collected on days 1, 3, 7, 14, 21, and 28 after force application (3 rats per group) using CO_2_ narcosis euthanasia for subsequent analysis.

### Establishment of a rat model of OTM with YAP-Inhibitor verteporfin (VP) treatment

Twenty-one rats, subjected to OTM with YAP-inhibitor verteporfin treatment (O + VP), received intraperitoneal injections of VP (100 mg/kg), starting 3 days before OTM and continuing every 2 days throughout the 28-day duration of OTM [32]. Mechanical force (25 g) was applied to the maxillary right first molar using a custom-made orthodontic nickel-titanium coil as previously described. After euthanasia using CO2 narcosis, specimens were collected on days 1, 3, 7, 14, 21, and 28 (3 rats per group) for further analysis (Fig. 6a).

### Extraction of total RNA from rat alveolar bone (in vivo study)

For total RNA extraction, four rats from each group subjected to 25 g force-induced tooth movement for 0, 1, and 3 days (the initial stage of OTM) were euthanized using CO2 narcosis. Hemimaxillae were dissected, cleaned, rinsed with sterile DEPC water, and flash-frozen in liquid nitrogen. Total RNA isolation followed established protocols [36]. Hemimaxillae were pulverized under liquid nitrogen, and RNA was extracted using Trizol (Invitrogen, Carlsbad, CA, USA) and RNeasy Mini Kits (Qiagen, Inc., Valencia, CA, USA). RNA underwent DNA removal with a DNA-free kit (Ambion, Austin, TX, USA), and its quality and quantity were assessed using a spectrophotometer (NanoDrop, Wilmington, DE, USA) and gel electrophoresis. The isolated total RNA was stored at -80 °C for subsequent analysis.

### RNA-Sequencing

The total RNA was performed RNA-Sequencing (RNA-seq) analysis to investigate the transcriptome. The Beijing Genome Institute (BGI) conducted transcriptome analysis, library preparation, and sequencing. Sequencing data were filtered using SOAPnuke (v1.5.2) to remove adapter contamination, reads with > 10% unknown base N content, and low-quality reads (base quality < 15 accounting for > 50% of the total bases in the read). Clean data were processed using the Dr. Tom Multi-omics Data Mining System (https://biosys.bgi.com). Alignment to the reference genome sequence was performed using HISAT (v2.1.0), and alignment to the reference gene sequence was done with Bowtie2 (v2.2.5), with gene expression levels calculated using RESM (v1.2.8). Known genes were annotated using seven databases, with transcription factor prediction. Mfuzz (v2.34.0) and WGCNA (v1.48) performed time series and co-expression network analysis. DESeq (Fold Change ≥ 2, Adjusted P-value ≤ 0.001) and PossionDis (Fold Change ≥ 2, FDR ≤ 0.001) conducted intra-group and inter-group differential gene analysis, respectively. Differential gene sets were clustered using pheatmap. Functional classification utilized GO and KEGG annotation results. KEGG and GO enrichment analyses were performed using the phyper function in R software (https://en.wikipedia.org/wiki/Hypergeometric_distribution) and TermFinder package (https://metacpan.org/pod/GO::TermFinder), respectively, with a Q-value ≤ 0.05 indicating significant enrichment. Further exploration of gene functions regarding phenotype changes included GO (http://www.geneontology.org/) and KEGG (https://www.kegg.jp/) enrichment analyses based on hypergeometric testing using Phyper, with the same Q-value threshold.

### Three-dimensional µCT morphometric analysis

After euthanasia, the maxillae were collected and fixed in 4% paraformaldehyde for 48 h. Subsequently, microfocus computed tomography (µCT, SkyScan 1174, Bruker) was utilized for scanning the maxillary samples, followed by three-dimensional (3D) reconstruction. The results were analyzed using lCT V6.0 software on the HP open platform (OpenVMS Alpha Version 1.3-1 session manager). On occlusal sections, three reference points (buccal embrasure, middle, and palatal embrasure) were identified on the distal surface of the first molar and mesial surface of the second molar at the height of the contour. The average distance of OTM between these points was quantitatively calculated. Random and systematic errors were assessed using previously described formulas [11]. The distance of OTM was depicted between the white dash lines in Fig. 1b.

### Cell culture studies

#### Establishment of a cell culture model of rat osteoclast precursors (ex-vivo)

Thirty SD rats were euthanized by CO2 narcosis to obtain osteoclast precursor cells (monocytes) from their femurs and tibiae. The bone marrow cavity was rinsed with PBS containing 5% penicillin-streptomycin (100 units/mL penicillin, 100 µg/mL streptomycin), and the collected wash solution underwent density gradient centrifugation using Lymphoprep™ (Accurate Chemical & Scientific Corporation, Westbury, NY, USA). The bone marrow derived-monocytes were isolated and purified from the mixed cells as described previously [37]. These monocytes were cultured in α-MEM medium with 5% fetal bovine serum and 1% penicillin-streptomycin at a density of 5 × 10^6^ cells/mL per well. Additionally, 20 ng/mL of macrophage colony-stimulating factor (M-CSF) and 20 ng/mL of receptor activator of nuclear factor kappa beta ligand (RANKL) were added to each well to induce pre-differentiation into osteoclast precursors. The cell culture was maintained at 37 °C with 5% CO_2_ and 95% air. After three days of incubation, the growth factors were removed from the cell culture, and the osteoclast precursors were then prepared for force application (Fig. 5a).

#### Application of compression

The osteoclast precursors were divided into five groups: the control group (no force applied) and four compression magnitude groups (0.5, 1, 1.5, and 2 g/cm^2^). Using a static compression loading system, various compression magnitudes were applied as previously described [38]. Briefly, a glass slide was positioned on the cells, and compression magnitudes were determined by calculating weight per unit area with stainless steel balls, using the calculation formula: Force per unit area of cells (g/cm^2^) = M /πr². The compression application lasted 24 h. Osteoclastogenesis was assessed using TRAP staining, while changes in YAP, Ctsk, and Col I expressions over time and their effects on signaling molecules under different compression magnitudes were examined (Fig. 5a).

#### Application of tension

The osteoclast precursors were divided into two groups: the control group (no force application) and the tension group. As previously described, isolated rat monocytes were cultured for 3 days on BioFlex^®^ stress cell culture plates (Flexcell International, Burlington, NC, USA) to induce pre-differentiation into osteoclast precursors. Tension was applied to the osteoclast precursors using an iStrain tension system, developed and patented by our laboratory [39]. The tension was applied at an extension rate of 12% and a frequency of 0.3 Hz of the culture membrane. Tension application lasted for 24 h, with intervals at 0, 6, 12, and 24 h. Osteoclastogenesis was evaluated by TRAP staining, and changes in YAP, Ctsk, and Col I expressions in osteoclasts over time, as well as the effects on signaling molecules under tension, were examined (Fig. 5a).

### Inhibition assay on YAP expression in rats’ osteoclasts (ex-vivo) under mechanical force (Compression/Tension)

Before applying compression/tension to the osteoclast precursors, verteporfin (VP) was added to inhibit YAP at a concentration of 10 µM per 1.5 ml. After 24 h of incubation with VP, compression or tension was applied to the osteoclast precursors according to the respective methods described above.

### Immunofluorescence staining

For the in vivo study, alveolar bone samples (including teeth) from the C and O groups were fixed in 4% paraformaldehyde for 48 hours. The samples were then decalcified using ethylenediaminetetraacetic acid (EDTA, pH 7.4), dehydrated in a gradient alcohol series, and cleared in xylene. After paraffin embedding, 5 µm thick serial sections were prepared and stained using immunofluorescence (IF). To detect YAP expression, the primary antibody YAP (1:50, Santa Cruz, sc-376830, USA) was applied and incubated overnight at 4°C. On the following day, specified secondary antibodies (goat anti-mouse IgG/RBITC or goat anti-mouse IgG/FITC from Solarbio) were applied at room temperature for 1 hour. Finally, cells were mounted using a medium containing 4’,6-diamidino-2-phenylindole (DAPI, Solarbio). Sections were observed and scanned with an Olympus^®^ microscope. Five random fields from each sample were selected for image analysis using ImageJ software.

### Immunohistochemistry

Immunohistochemistry (IHC) was used to observe the expressions of Ctsk and Col I in the alveolar bone under mechanical force (compression/tension) during OTM. Sections were blocked with a buffer containing 1× PBS, 5% BSA, and 0.3% Triton™ X-100. They were then incubated overnight at 4 °C with primary antibodies against Ctsk (1:50, Santa Cruz, sc-48353, USA) and Col I (1:500, Abcam, ab270993, CB, UK). Following this, sections were incubated with an HRP-conjugated goat anti-mouse IgG secondary antibody (1:200, Solarbio, SF131) at room temperature for 1–2 h. Finally, sections were mounted with neutral resin. Imaging was captured using an Olympus^®^ microscopy imaging system. Five random fields from each sample were selected, and image analysis was performed using ImageJ software.

### TRAP staining

To observe osteoclast formation during in vivo alveolar bone remodeling under mechanical force and the effect of mechanical force (compression/tension) on the differentiation of osteoclast precursors (monocytes, ex-vivo), tartrate-resistant acid phosphatase (TRAP) staining was performed. The staining was conducted using the TRAP staining kit (Sigma-Aldrich, 387 A, USA) according to the provided instructions.

### Western blot

At 0, 6, 12 and 24 h, cells from each group were collected and treated with RIPA buffer (pH 7.0) (Sigma-Aldrich, Inc. USA) containing a Halt™ protease and phosphatase inhibitor cocktail, EDTA-free (1:100, 78441, Thermo Scientific™, USA). Total protein was extracted by centrifugation after completely lysis, and protein concentrations determined using a bicinchoninic acid (BCA) protein assay kit (Thermo Fisher Scientific, Inc., USA). Samples were performed sodium dodecyl sulfate–polyacrylamide gel electrophoresis (SDS-PAGE) and transferred to nitrocellulose membranes (Millipore, Billerica, MA, USA). Membranes were blocked with 5% bovine serum albumin (BSA) in TBST for 1 h, then incubated overnight at 4 ℃ with primary antibodies: YAP (1:200, Santa Cruz, sc-376830, USA), TEAD (1:500, Proteintech, 13283-1-AP, IL, USA), LATS (1:200, Santa Cruz, sc-398560, USA), NF-κB p65 (1:500, Santa Cruz, sc-8008, USA), and RANK (1:500, Santa Cruz, sc-374360, USA). After incubation, species-specific secondary antibodies, either goat anti-mouse IgG-HRP (1:5000, Solarbio, SE131) or goat anti-rabbit IgG-HRP (1:5000, Solarbio, SE134), were applied and incubated at room temperature for 1 h. Protein detection used SuperSignal™ West Dura Extended Duration Substrate(Thermo Scientific, 34075, USA), with β-actin as an internal control. Membranes were scanned with the BIO-RAD ChemiDocTM MP Imaging System (BIO-RAD, CA, USA) for imaging and documentation of western blot bands, and densitometric units measured using Image J analysis software.

### Gelatin zymography for MMP-9 analysis

At 0, 6, 12, and 24 h, the conditioned media from each group of cells were collected and analyzed for pro- (92 kDa) and active- (82 kDa) MMP-9 by gelatin zymography as described previously [40–43]. The clear zones of gelatin lysis against a background indicated gelatinolytic activity and were scanned densitometrically with the BIO-RAD ChemiDoc™ MP Imaging System (BIO-RAD, CA, USA). Results were analyzed by Image J to quantitatively assess gelatinase activity [44]. MMP-9 standard was purchased from R&D Systems, Inc. (Minneapolis, MN, USA).

### Statistics

The data were analyzed using SPSS 28.0.1 (IBM) software. The analysis included both analysis of variance (*ANOVA*) and Student’s *t-test*, conducted separately by two investigators, with significance set at *p* < 0.05. In vivo data are presented as mean ± standard error (S.E.M.) (*n* = 3 ~ 5/group), while data from cell cultures are presented as mean ± S.E.M. (*n* = 4/group). Experiments were repeated independently at least three times.

## Electronic supplementary material

Below is the link to the electronic supplementary material.

Supplementary Material 1

Supplementary Material 2

Supplementary Material 3

Supplementary Material 4

## Data Availability

All data and other supporting information is available upon request from the corresponding author.

## Abbreviations

ANOVA: Analysis included both analysis of variance
BCA: Bicinchoninic acid
BGI: Beijing Genome Institute
BMUs: Basic multicellular units
BSA: Bovine serum albumin
C group: Control group
Col I: Type I collagen
Ctsk: Cathepsin K
DAPI: 4’,6-diamidino-2-phenylindole
DEPC water: Deionized, diethylpyrocarbonate treated water
EDTA: Ethylenediaminetetraacetic acid
IF: Immunofluorescence
IHC: Immunohistochemistry
i.p.: intraperitoneal
KEGG pathway: Kyoto encyclopedia of genes and genomes pathway
LATS1/2: Large tumor suppressor kinase 1/2
M-CSF: Macrophage colony-stimulating factor
µCT: Microfocus computed tomography
MST1/2: Mammalian sterile 20-like protein kinase 1/2
NF-κB: Nuclear factor kappa B
O group: Orthodontic tooth movement group
O + VP group: OTM with YAP-inhibitor verteporfin treatment
OBs: Osteoblasts
OCs: Osteoclasts
OTM: Orthodontic tooth movement
PDL: Periodontal ligament
RANK: Receptor activator of nuclear factor kappa B
RANKL: Receptor activator of nuclear factor kappa B ligand
RNA-seq: RNA-Sequencing
SD rats: Sprague Dawley Rats
SDS-PAGE: Sodium dodecyl sulfate–polyacrylamide gel electrophoresis
S.E.M.: Standard error
TAZ: Transcriptional coactivator with a PDZ-binding motif
TEAD1-4: Transcriptional enhancer factor domain family member 1–4
3D: Three-dimensional
TRAP: Tartrate-resistant acid phosphatase
TRAP^+^ OCs: TRAP staining positive osteoclasts
VP: Verteporfin
YAP: Yes-associated protein

## References

[1] Bolamperti S, Villa I, Rubinacci A. Bone remodeling: an operational process ensuring survival and bone mechanical competence. Bone Res. 2022;10(1):48. 35851054 10.1038/s41413-022-00219-8 PMC9293977

[2] Sims NA, Martin TJ. Osteoclasts provide coupling signals to osteoblast lineage cells through multiple mechanisms. Annu Rev Physiol. 2020;82:507–29. 31553686 10.1146/annurev-physiol-021119-034425

[3] Chen X, Wang Z, Duan N, Zhu G, Schwarz EM, Xie C. Osteoblast-osteoclast interactions. Connect Tissue Res. 2018;59(2):99–107. 28324674 10.1080/03008207.2017.1290085 PMC5612831

[4] Matsuo K, Irie N. Osteoclast-osteoblast communication. Arch Biochem Biophys. 2008;473(2):201–9. 18406338 10.1016/j.abb.2008.03.027

[5] Daponte V, Henke K, Drissi H. Current perspectives on the multiple roles of osteoclasts: mechanisms of osteoclast-osteoblast communication and potential clinical implications. Elife. 2024;13. 10.7554/eLife.95083 PMC11003748 38591777

[6] Lassen NE, Andersen TL, Ploen GG, Soe K, Hauge EM, Harving S, et al. Coupling of bone resorption and formation in Real Time: New Knowledge gained from human haversian BMUs. J Bone Min Res. 2017;32(7):1395–405. 10.1002/jbmr.3091 28177141

[7] Arias CF, Herrero MA, Echeverri LF, Oleaga GE, Lopez JM. Bone remodeling: a tissue-level process emerging from cell-level molecular algorithms. PLoS ONE. 2018;13(9):e0204171. 30231062 10.1371/journal.pone.0204171 PMC6145577

[8] Udagawa N, Koide M, Nakamura M, Nakamichi Y, Yamashita T, Uehara S, et al. Osteoclast differentiation by RANKL and OPG signaling pathways. J Bone Min Metab. 2021;39(1):19–26. 10.1007/s00774-020-01162-6 33079279

[9] Yu L, Zhang XR, Deng J. Quantitative Histology of Response of Sutures under Retractive Force with Zygomatic Implant Anchorage to the Maxilla of Rhesus Monkey. Acta Med Univ Sci Technol Huazhong. 2014;43(1):39–44.

[10] Deng J, Beroukhim J, Parizadeh P, Shembesh FA. Response of Mid-Palatal Suture to Compressive Stress. 91st European Orthodontic Society (EOS); June; Venice, Italy2015.

[11] Alikhani M, Alyami B, Lee IS, Almoammar S, Vongthongleur T, Alikhani M, et al. Saturation of the biological response to orthodontic forces and its effect on the rate of tooth movement. Orthod Craniofac Res. 2015;18(Suppl 1):8–17. 25865529 10.1111/ocr.12090

[12] Piccolo S, Dupont S, Cordenonsi M. The biology of YAP/TAZ: hippo signaling and beyond. Physiol Rev. 2014;94(4):1287–312. 25287865 10.1152/physrev.00005.2014

[13] Dupont S, Morsut L, Aragona M, Enzo E, Giulitti S, Cordenonsi M, et al. Role of YAP/TAZ in mechanotransduction. Nature. 2011;474(7350):179–83. 21654799 10.1038/nature10137

[14] Aragona M, Panciera T, Manfrin A, Giulitti S, Michielin F, Elvassore N, et al. A mechanical checkpoint controls multicellular growth through YAP/TAZ regulation by actin-Processing factors. Cell. 2013;154(5):1047–59. 23954413 10.1016/j.cell.2013.07.042

[15] Sun B, Wen Y, Wu X, Zhang Y, Qiao X, Xu X. Expression pattern of YAP and TAZ during orthodontic tooth movement in rats. J Mol Histol. 2018;49(2):123–31. 29356923 10.1007/s10735-017-9752-1

[16] Heidary Arash E, Shiban A, Song S, Attisano L. MARK4 inhibits Hippo signaling to promote proliferation and migration of breast cancer cells. EMBO Rep. 2017;18(3):420–36. 28183853 10.15252/embr.201642455 PMC5331264

[17] Meng Z, Moroishi T, Guan KL. Mechanisms of Hippo pathway regulation. Genes Dev. 2016;30(1):1–17. 26728553 10.1101/gad.274027.115 PMC4701972

[18] Deng J, Zhang XR. Histomorphological changes during remodeling of Premaxillary suture and Zygomaticomaxillary suture under different mechanical forces in rabbits. Acta Med Univ Sci Technol Huazhong. 2015;44(2):165–70.

[19] Loundagin LL, Harrison KD, Wei X, Cooper DML. Understanding basic multicellular unit activity in cortical bone through 3D morphological analysis: new methods to define zones of the remodeling space. Bone. 2024;179:116960. 37972746 10.1016/j.bone.2023.116960

[20] Bhatt HD, McClain S, Lee HM, Deng J, Johnson F, Gu Y et al. The maximum-tolerated-dose and pharmacokinetics of a Novel chemically-modified-curcumin in rats. J Dent Res. 2020;99(Special Issue A):3665. 10.2147/JEP.S341927 PMC8842656 35173493

[21] Crane JL, Xian L, Cao X. Role of TGF-beta signaling in coupling bone remodeling. Methods Mol Biol. 2016;1344:287–300. 26520132 10.1007/978-1-4939-2966-5_18

[22] Furuya M, Kikuta J, Fujimori S, Seno S, Maeda H, Shirazaki M, et al. Direct cell-cell contact between mature osteoblasts and osteoclasts dynamically controls their functions in vivo. Nat Commun. 2018;9(1):300. 29352112 10.1038/s41467-017-02541-w PMC5775424

[23] Henriksen K, Karsdal MA, Martin TJ. Osteoclast-derived coupling factors in bone remodeling. Calcif Tissue Int. 2014;94(1):88–97. 23700149 10.1007/s00223-013-9741-7

[24] Martin TJ, Sims NA. Osteoclast-derived activity in the coupling of bone formation to resorption. Trends Mol Med. 2005;11(2):76–81. 15694870 10.1016/j.molmed.2004.12.004

[25] Sims NA, Martin TJ. Coupling the activities of bone formation and resorption: a multitude of signals within the basic multicellular unit. Bonekey Rep. 2014;3:481. 24466412 10.1038/bonekey.2013.215 PMC3899560

[26] Zhao L, Guan H, Song C, Wang Y, Liu C, Cai C, et al. YAP1 is essential for osteoclastogenesis through a TEADs-dependent mechanism. Bone. 2018;110:177–86. 29432919 10.1016/j.bone.2018.01.035

[27] Yang W, Han W, Qin A, Wang Z, Xu J, Qian Y. The emerging role of Hippo signaling pathway in regulating osteoclast formation. J Cell Physiol. 2018;233(6):4606–17. 29219182 10.1002/jcp.26372

[28] Li S, Li Q, Zhu Y, Hu W. GDF15 induced by compressive force contributes to osteoclast differentiation in human periodontal ligament cells. Exp Cell Res. 2020;387(1):111745. 31765611 10.1016/j.yexcr.2019.111745

[29] Donohue E, Tovey A, Vogl AW, Arns S, Sternberg E, Young RN, et al. Inhibition of autophagosome formation by the benzoporphyrin derivative verteporfin. J Biol Chem. 2011;286(9):7290–300. 21193398 10.1074/jbc.M110.139915 PMC3044985

[30] Wei C, Li X. Verteporfin inhibits cell proliferation and induces apoptosis in different subtypes of breast cancer cell lines without light activation. BMC Cancer. 2020;20(1):1042. 33121449 10.1186/s12885-020-07555-0 PMC7599100

[31] Saini H, Sharma H, Mukherjee S, Chowdhury S, Chowdhury R. Verteporfin disrupts multiple steps of autophagy and regulates p53 to sensitize osteosarcoma cells. Cancer Cell Int. 2021;21(1):52. 33446200 10.1186/s12935-020-01720-y PMC7807844

[32] Gavini J, Dommann N, Jakob MO, Keogh A, Bouchez LC, Karkampouna S, et al. Verteporfin-induced lysosomal compartment dysregulation potentiates the effect of sorafenib in hepatocellular carcinoma. Cell Death Dis. 2019;10(10):749. 31582741 10.1038/s41419-019-1989-z PMC6776510

[33] Ma S, Meng Z, Chen R, Guan KL. The Hippo Pathway: Biology and Pathophysiology. Annu Rev Biochem. 2019;88:577–604. 30566373 10.1146/annurev-biochem-013118-111829

[34] Panciera T, Azzolin L, Cordenonsi M, Piccolo S. Mechanobiology of YAP and TAZ in physiology and disease. Nat Rev Mol Cell Biol. 2017;18(12):758–70. 28951564 10.1038/nrm.2017.87 PMC6192510

[35] Percie du Sert N, Ahluwalia A, Alam S, Avey MT, Baker M, Browne WJ, et al. Reporting animal research: explanation and elaboration for the ARRIVE guidelines 2.0. PLoS Biol. 2020;18(7):e3000411. 32663221 10.1371/journal.pbio.3000411 PMC7360025

[36] Mantila Roosa SM, Liu Y, Turner CH. Gene expression patterns in bone following mechanical loading. J Bone Min Res. 2011;26(1):100–12. 10.1002/jbmr.193 PMC3179310 20658561

[37] Liao G, Simone J, Simon SR. Paracrine downregulation of fc gamma RIII in human monocyte-derived macrophages induced by phagocytosis of nonopsonized particles. Blood. 1994;83(8):2294–304. 8161796

[38] He D, Kou X, Yang R, Liu D, Wang X, Luo Q, et al. M1-like macrophage polarization promotes orthodontic tooth Movement. J Dent Res. 2015;94(9):1286–94. 26124217 10.1177/0022034515589714

[39] Zhao Y, Huang Y, Jia L, Wang R, Tan K, Li W. A novel tension machine promotes bone marrow mesenchymal stem cell osteoblastic and fibroblastic differentiation by applying cyclic tension. Stem Cells Int. 2021;2021:6647651. 34422062 10.1155/2021/6647651 PMC8371653

[40] Golub LM, Ramamurthy NS, Llavaneras A, Ryan ME, Lee HM, Liu Y, et al. A chemically modified nonantimicrobial tetracycline (CMT-8) inhibits gingival matrix metalloproteinases, periodontal breakdown, and extra-oral bone loss in ovariectomized rats. Ann N Y Acad Sci. 1999;878:290–310. 10415737 10.1111/j.1749-6632.1999.tb07691.x

[41] Lee HM, Golub LM, Cao J, Teronen O, Laitinen M, Salo T, et al. CMT-3, a non-antimicrobial tetracycline (TC), inhibits MT1-MMP activity: relevance to cancer. Curr Med Chem. 2001;8(3):257–60. 11172680 10.2174/0929867013373660

[42] Elburki MS. A Novel chemically modified Curcumin as a pleiotropic MMP-Inhibitor: therapeutic potential in locally- and systemically-Induced Periodontal (and other). Connective tissue breakdown. University Libraries on behalf of the Graduate School at Stony Brook University. Stony Brook University; 2015.

[43] Elburki MS, Moore DD, Terezakis NG, Zhang Y, Lee HM, Johnson F, et al. A novel chemically modified curcumin reduces inflammation-mediated connective tissue breakdown in a rat model of diabetes: periodontal and systemic effects. J Periodontal Res. 2017;52(2):186–200. 27038334 10.1111/jre.12381

[44] Elburki MS, Rossa C Jr., Guimaraes-Stabili MR, Lee HM, Curylofo-Zotti FA, Johnson F, et al. A chemically modified Curcumin (CMC 2.24) inhibits nuclear factor kappaB activation and inflammatory bone loss in murine models of LPS-Induced Experimental Periodontitis and Diabetes-Associated Natural Periodontitis. Inflammation. 2017;40(4):1436–49. 28534138 10.1007/s10753-017-0587-4

