# Supplementary material for: Mechanosensor YAP mediates bone remodeling via NF-κB p65 induced osteoclastogenesis during orthodontic tooth movement
Source: Prog Orthod. 2025 Jan 2;26:2. doi: 10.1186/s40510-024-00548-w (PMC11695529; doi:10.1186/s40510-024-00548-w)
Supplement: Supplementary file 1 — Supplementary Material 1 [file 40510_2024_548_MOESM1_ESM.docx]

Supplementary Material

Mechanosensor YAP Mediates Bone Remodeling via NF-κB p65 Induced Osteoclastogenesis During Orthodontic Tooth Movement

Jie Deng^1,2^, Yu-Ning Zhang^1^, Ru-Shui Bai^1^, Ting-Ting Yu^1^, Yi Zhao^1^, Hao Liu^1^, Yun-Fan Zhang^1^, Tian-Min Xu^1*^, and Bing Han^1,3*^

**Figure Legends**

Figure S1. The variation of YAP expression, osteoclastogenesis, and Col I expression over time under mechanical force (compression/tension) (25g) from day 0 to day 28 of OTM. (a) Histological changes over time in the expression of YAP (IF), quantity of TRAP^+^ OCs, and expressions of Ctsk and Col I (IHC) on the compression side of alveolar bone under mechanical force from day 0 to day 28. (b) Histological changes over time in the expression of YAP (IF), quantity of TRAP^+^ OCs, and expressions of Ctsk and Col I (IHC) on the tension side of alveolar bone under mechanical force from day 0 to day 28. The images were shown with 20× magnification, and high magnification (40×) for indicating TRAP^+^ OCs and the Ctsk expressions.

Figure S2. The changes in YAP expression over time (6 and 12 hours) in rat OCs under different compression magnitudes (0.5, 1, 1.5, 2g) are depicted. The YAP expressions in rat OCs were examined by IF. The images were shown with 40× magnification.

Figure S3. The IF staining images of YAP inhibition and TRAP staining in the alveolar bone over time (from day 0 to 28) with VP injection under mechanical force (compression/tension) are presented. (a) Histological changes over time in the expression of YAP (IF) and quantity of TRAP^+^ OCs on the compression side of alveolar bone with VP injection under mechanical force from day 0 to day 28. (b) Histological changes over time in the expression of YAP (IF) and quantity of TRAP^+^ OCs on the tension side of alveolar bone with VP injection under mechanical force from day 0 to day 28. The images were shown with 20× magnification, and high magnification (40×) for indicating TRAP^+^ OCs.


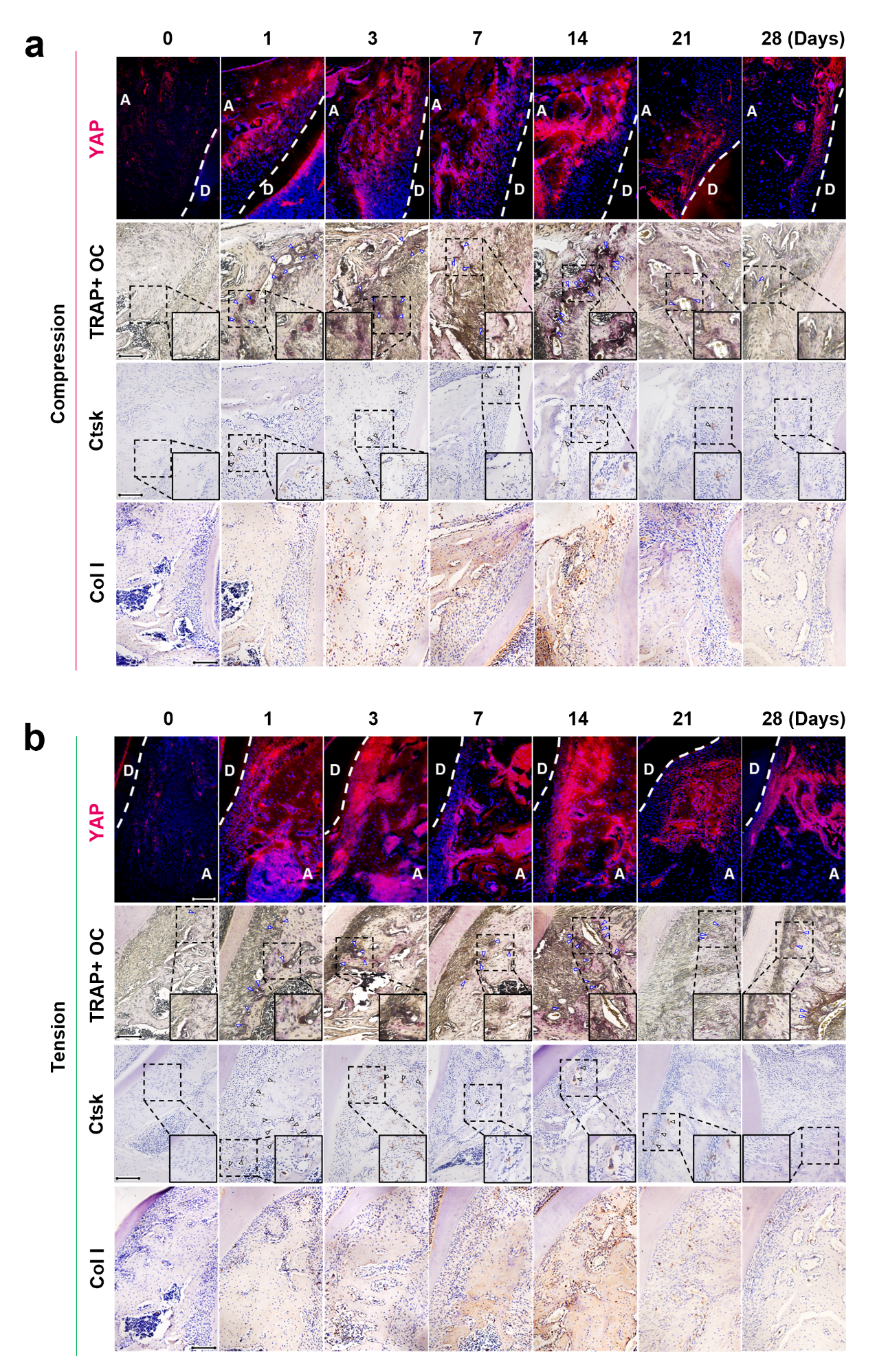


Figure S1.


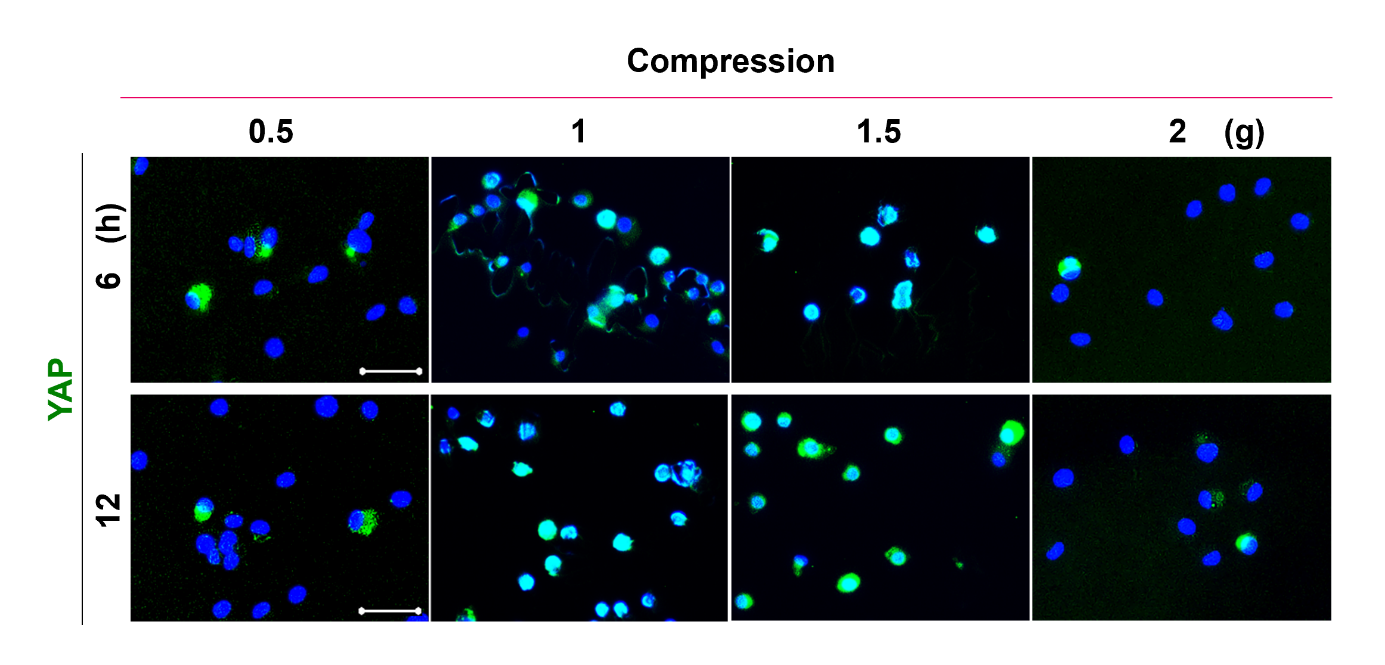


Figure S2.


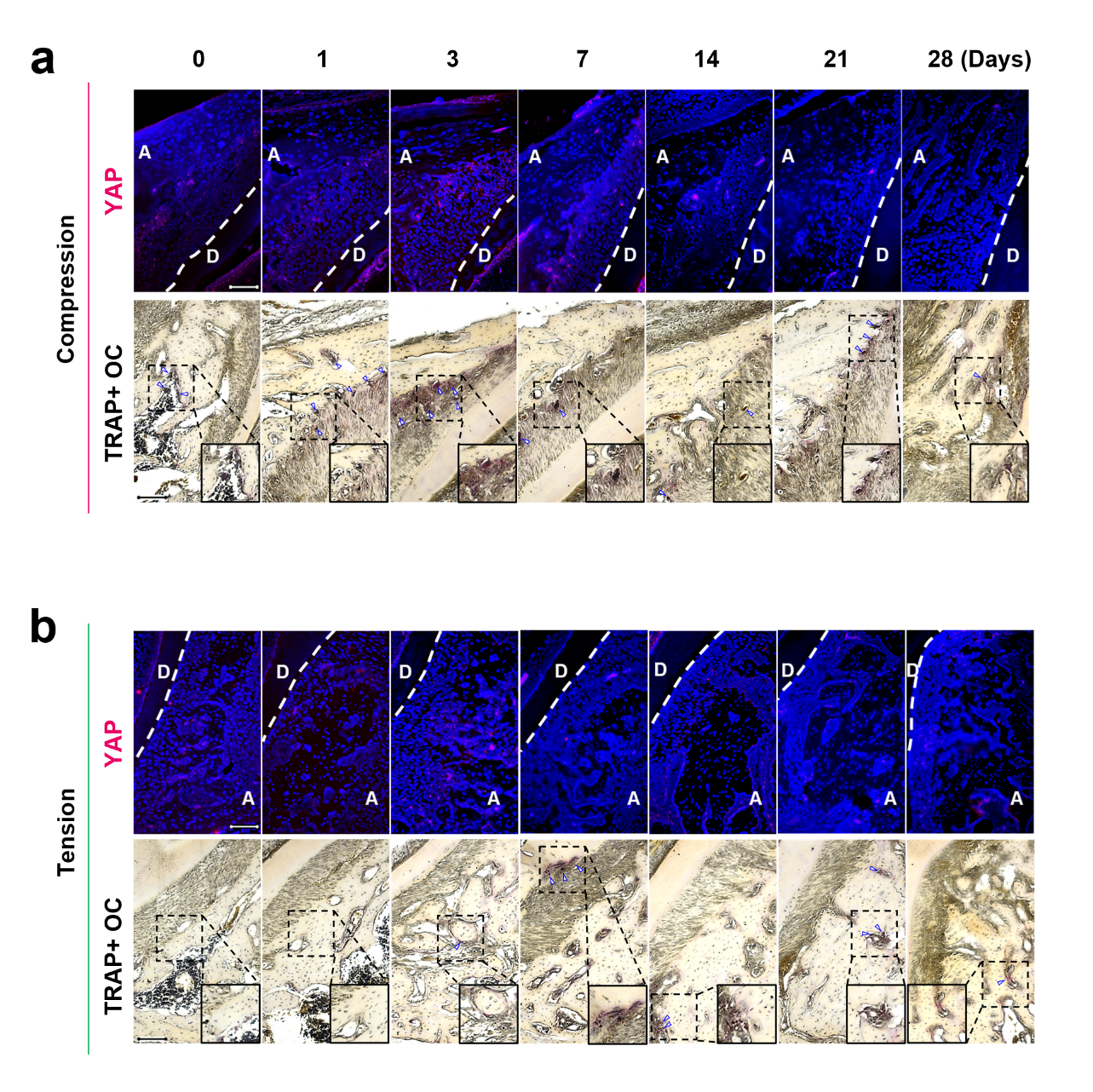


Figure S3.
