# Supplementary figures and images for: Mechanosensor YAP mediates bone remodeling via NF-κB p65 induced osteoclastogenesis during orthodontic tooth movement
Source: Prog Orthod. 2025 Jan 2;26:2. doi: 10.1186/s40510-024-00548-w (PMC11695529; doi:10.1186/s40510-024-00548-w)

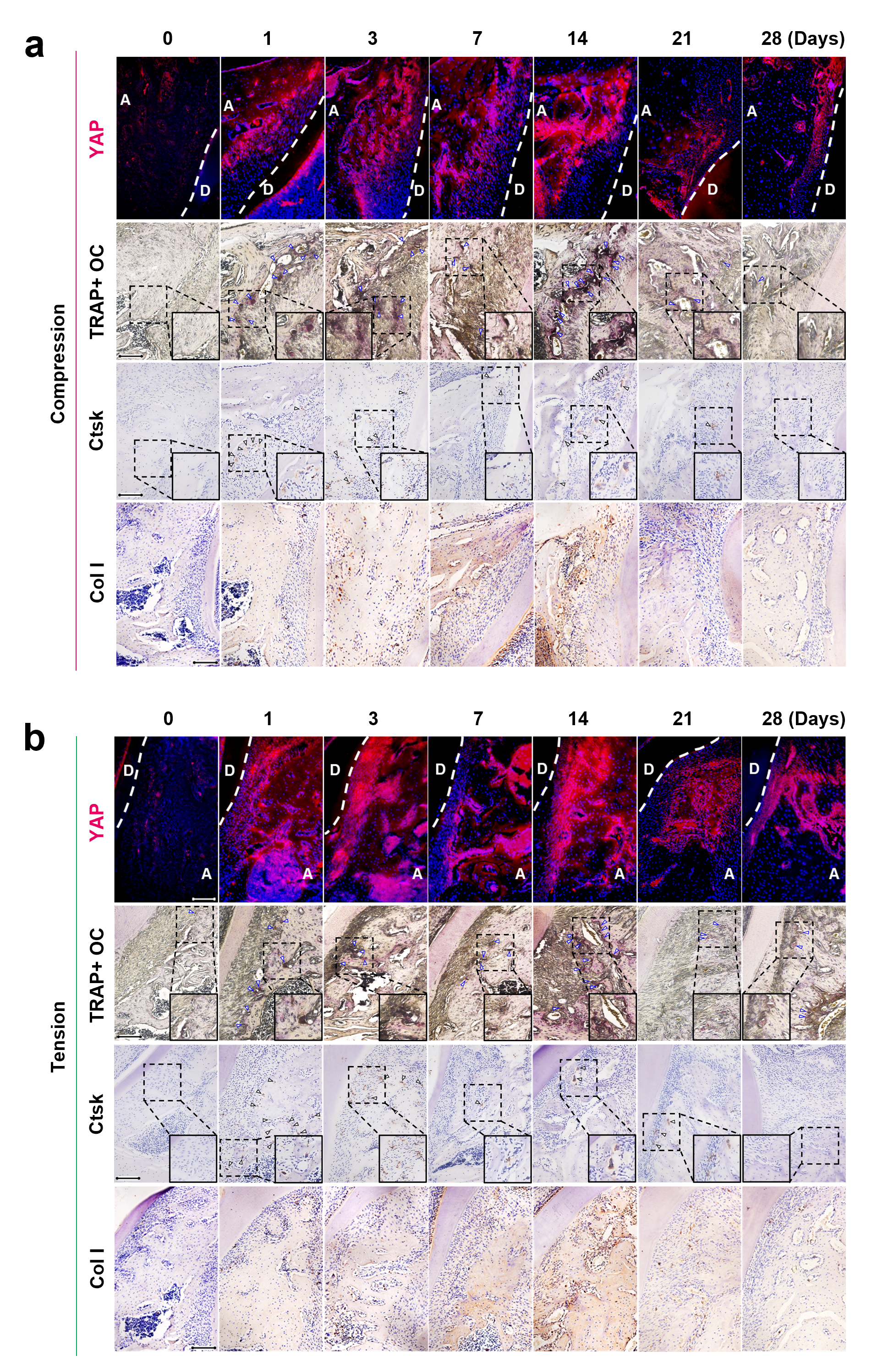

Supplement: Supplementary file 2 — Supplementary Material 2 [file 40510_2024_548_MOESM2_ESM.tif]

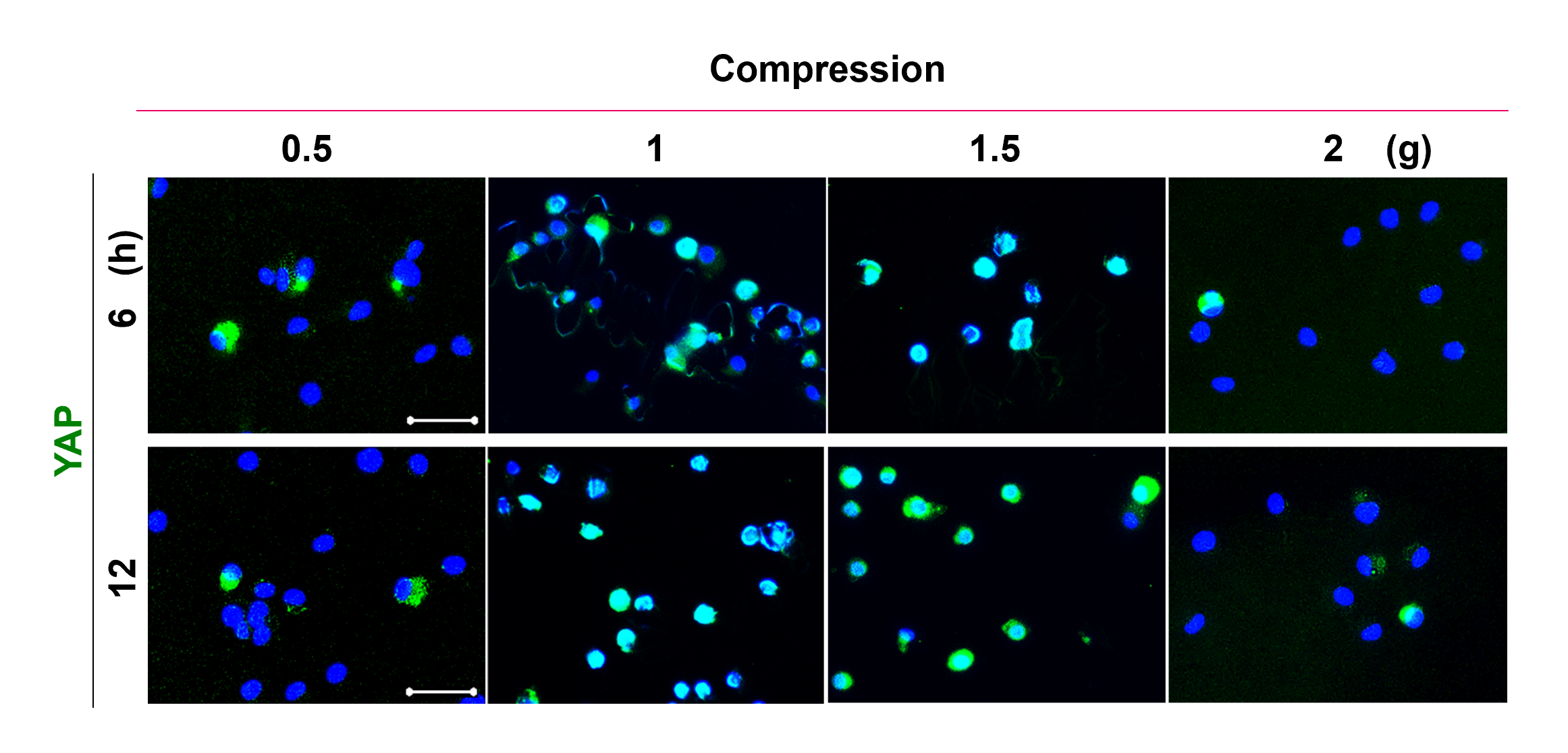

Supplement: Supplementary file 3 — Supplementary Material 3 [file 40510_2024_548_MOESM3_ESM.tif]

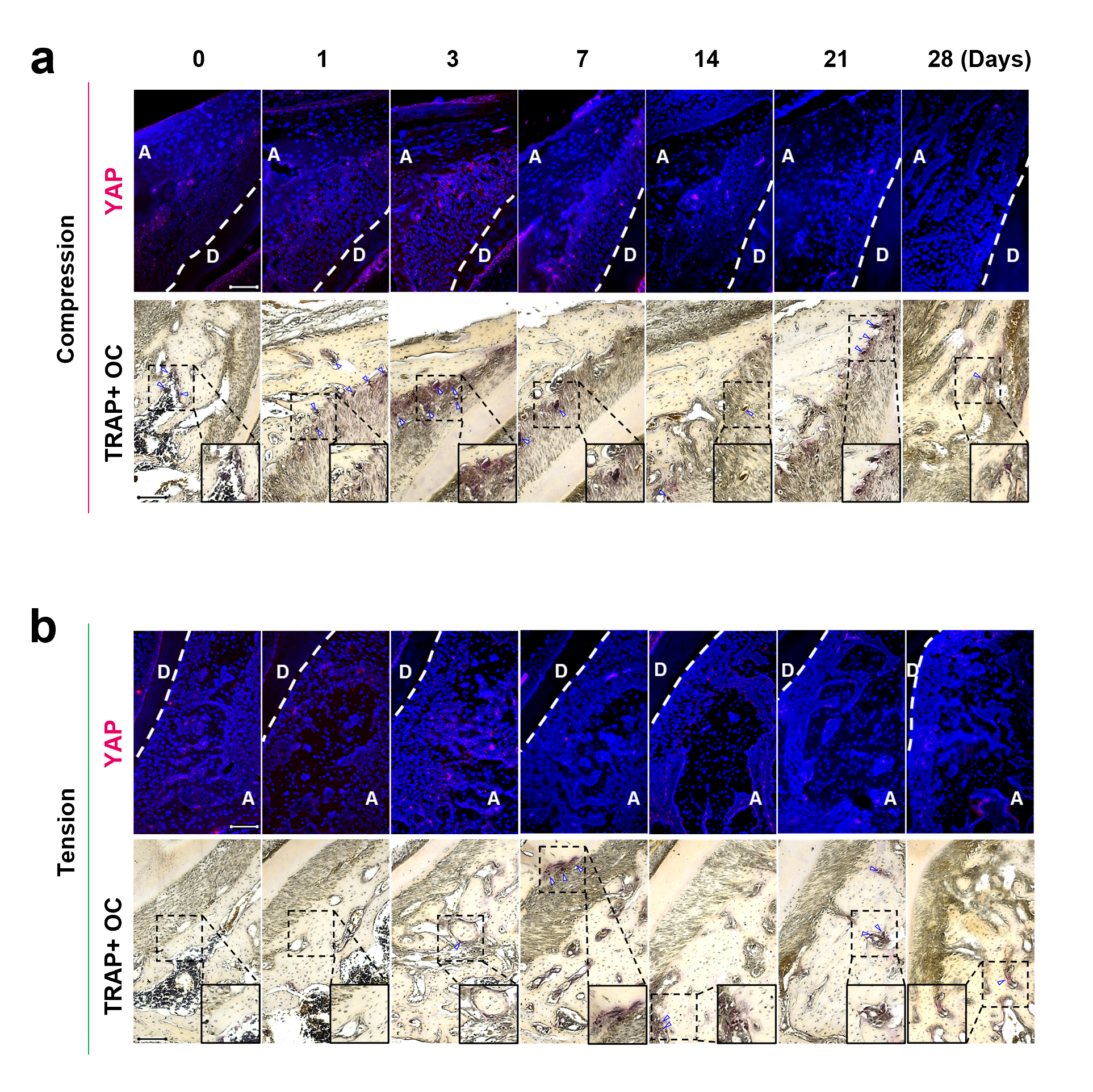

Supplement: Supplementary file 4 — Supplementary Material 4 [file 40510_2024_548_MOESM4_ESM.tif]
